# SARS-CoV-2 spike antibodies cross-react with dengue virus and enhance infection *in vitro* and *in vivo*

**DOI:** 10.3389/fimmu.2025.1724625

**Published:** 2026-01-15

**Authors:** Kamini Jakhar, Sudipta Sonar, Gagandeep Singh, Tejeswara Rao Asuru, Garima Joshi, Nisha Beniwal, Tania Sarkar, Mahima Tiwari, Jaskaran Kaur, Deepak Kumar Rathore, Banwari Lal, Sandeep Kumar, Puneet Srivastav, Satendra Kumar, Vikas Phagna, Sushma Mithina, Lokesh Kumar, Vishal Gupta, Pallavi Kshetrapal, Savita Singh, Nitya Wadhwa, Ramachandran Thiruvengadam, Sreevatsan Raghavan, Mudita Gosain, Tripti Shrivastava, Sankar Bhattacharyya, Shailendra Asthana, Prasenjit Guchhait, Shailendra Mani

**Affiliations:** 1 Centre for Virus Research, Therapeutics and Vaccines, Translational Health Science and Technology Institute, Faridabad, India; 2 Computational Biophysics and Computer-Aided Drug Design (CADD) Group, Computational and Mathematical Biology Centre (CMBC), Translational Health Science and Technology Institute (THSTI), Faridabad, India; 3 Molecular Medicine, Regional Centre for Biotechnology, Faridabad, India; 4 Centre for Immunobiology and Immunotherapy, Translational Health Science and Technology Institute, Faridabad, India; 5 Translational Health Science and Technology Institute, Faridabad, India; 6 Centre for Maternal and Child Health, Translational Health Science and Technology Institute, Faridabad, India

**Keywords:** AG129 mice model, antibody-dependent enhancement, cross-reactivity, dengue virus, SARS-CoV-2

## Abstract

The presence of non-neutralizing antibodies of any dengue serotype increases the severity of subsequent infection by other dengue serotypes. During the SARS-CoV-2 pandemic, the number of symptomatic dengue cases increased in India. We found that antibodies isolated from convalescent plasma from COVID-19 patients enhances DENV2 infection *in vitro*. CR3022, an antibody against the SARS-CoV-2 spike protein, also elevated DENV2 infection *in vitro*. *In silico* protein–protein interactions between spike antibodies and the DENV2 E-protein revealed significant interactions. Likewise, few monoclonal/polyclonal antibodies against SARS-CoV-2 have shown increased dengue infection *in vitro*. Importantly, AG129 mice infected with SARS-CoV-2 three weeks prior to DENV2 infection showed elevated dengue pathogenesis. This highlights the possibility of elevated infection and symptomatic dengue disease in COVID-19 survivors.

## Introduction

Dengue is endemic in more than 120 countries, with Asia contributing to 70% of the global burden (1). Dengue virus (DENV) circulates in four serotypes (DENV 1–4), each containing multiple distinct genotypes. Primary infection provides lifelong immunity to homotypic secondary infections but provides partial immunity to heterotypic challenges. Heterotypic secondary infections present severe symptoms due to higher viremia catalyzed by antibody-dependent enhancement (ADE). Sub-neutralizing antibodies that bind to heterotypic DENV promote FcR-mediated viral entry into cells (2). A stable attachment of these antibodies to FC receptors, without reaching the neutralization threshold, leads to ADE (3).

Dengue serotypes and their severity vary across different regions. Although all four types (DENV1–4) can cause anything from mild fever to hemorrhagic shock, recent data show that DENV2 and DENV3 account for most of the severe cases. During the 2023–2024 dengue seasons in South and Southeast Asia, approximately 57% of dengue cases in Bangladesh were DENV3, with DENV2 accounting for approximately 30% (4) Surveillance in Puerto Rico similarly observed DENV3 replacing DENV1 as the dominant serotype in 2023–2024, and found that infections from DENV2 or DENV3 led to higher hospitalization and severe disease rates than DENV1 (5). In Mexico, a retrospective study strongly linked DENV2 with severe outcomes, while DENV4 seemed to have a protective effect (6). A 19−year pediatric cohort in Nicaragua further showed that DENV2 and DENV3 produced the highest proportion of severe cases according to WHO classifications, with DENV2 causing more severe illness during secondary infections and being associated with pleural effusion and thrombocytopenia, whereas DENV3 was linked to hypotensive and compensated shock (7). In India, secondary infections with DENV2 are most often associated with severe disease, while DENV4 is less commonly linked to severe cases (8).

The dynamics of DENV infections during the SARS-CoV-2 pandemic varied globally. A 16% decline in dengue incidence was reported in 2020–2021 compared to pre-COVID-19 (9). However, Khan et al. revealed a substantial surge in dengue cases in 2021 in specific South Asian countries, including India (~3-fold), Pakistan (>7-fold), and Bangladesh (>19-fold). This contrasts with a noteworthy decline of approximately 36% across Southeast Asia and Latin America (10). Several regional, environmental, and pandemic-related elements have shaped these intricate dynamics. Disruptions in routine mosquito vector surveillance and control programs during COVID-19 lockdowns may have led to a rise in dengue cases, highlighting the crucial role of vector control in preventing the disease. The similar pathologies of DENV and COVID-19 present diagnostic challenges, potentially contributing to underreporting (9, 11–14).

Some studies have indicated that ADE due to prior SARS-CoV-2 infection may elevate the risk of symptomatic and/or severe dengue. In Pakistan, a significant increase in dengue-related deaths (linked to DENV-2 prevalence) was observed in patients with pre-exposure to SARS-CoV-2 (15).

Modest serological cross-reactivity between SARS-CoV-2 and DENV-2 has been reported (16–18). However, it is unclear whether this would have any clinical impact on dengue cases (19). A study reported that rabbit IgG against purified SARS-CoV-2 S1-RBD cross-reacted with DENV Envelope (E), precursor-membrane (PrM), and non-structural protein 1 (NS1). However, these antibodies did not enhance DENV infection in THP-1 cells expressing FcR. Moreover, they inhibited DENV infection at high concentrations (20). Another study showed *in vitro* inhibition of dengue infection by sera from convalescent COVID-19 patients (21).

Nath et al. demonstrated serological cross-reactivity of SARS-CoV-2 positive serum samples with DENV1 and that the sera neutralized DENV1 (21). Anti-S1-RBD antibodies were shown to impede DENV infection at a specific concentration and COVID-19 patients with sera exhibited a neutralizing ability against dengue infection (20). El-Qushayri et al., in their systematic review, aimed to assess the clinical outcomes of patients with dengue and COVID-19 co-infection. They reported a high mortality rate in co-infected patients (19.1%), significantly exceeding the estimated global mortality rates for dengue (1.3%) and COVID-19 (2.04%). They also found that the hospital stay duration for co-infected individuals was longer than the reported median stays for standalone COVID-19 and dengue cases (22). Although insightful, more information on the stoichiometry of the virus and antibodies in these studies would have been useful since it is crucial for understanding antibody–virus interactions and the potential for ADE.

Here, we evaluated the effect of preexisting SARS-CoV-2 antibodies on DENV-2 infection using cell-based assays and a mouse model of DENV infection.

## Results

### *In silico* interaction mining drives the cross-recognition of DENV-E protein by anti-spike and anti-RBD SARS-CoV-2 antibodies

**Figure 1:**
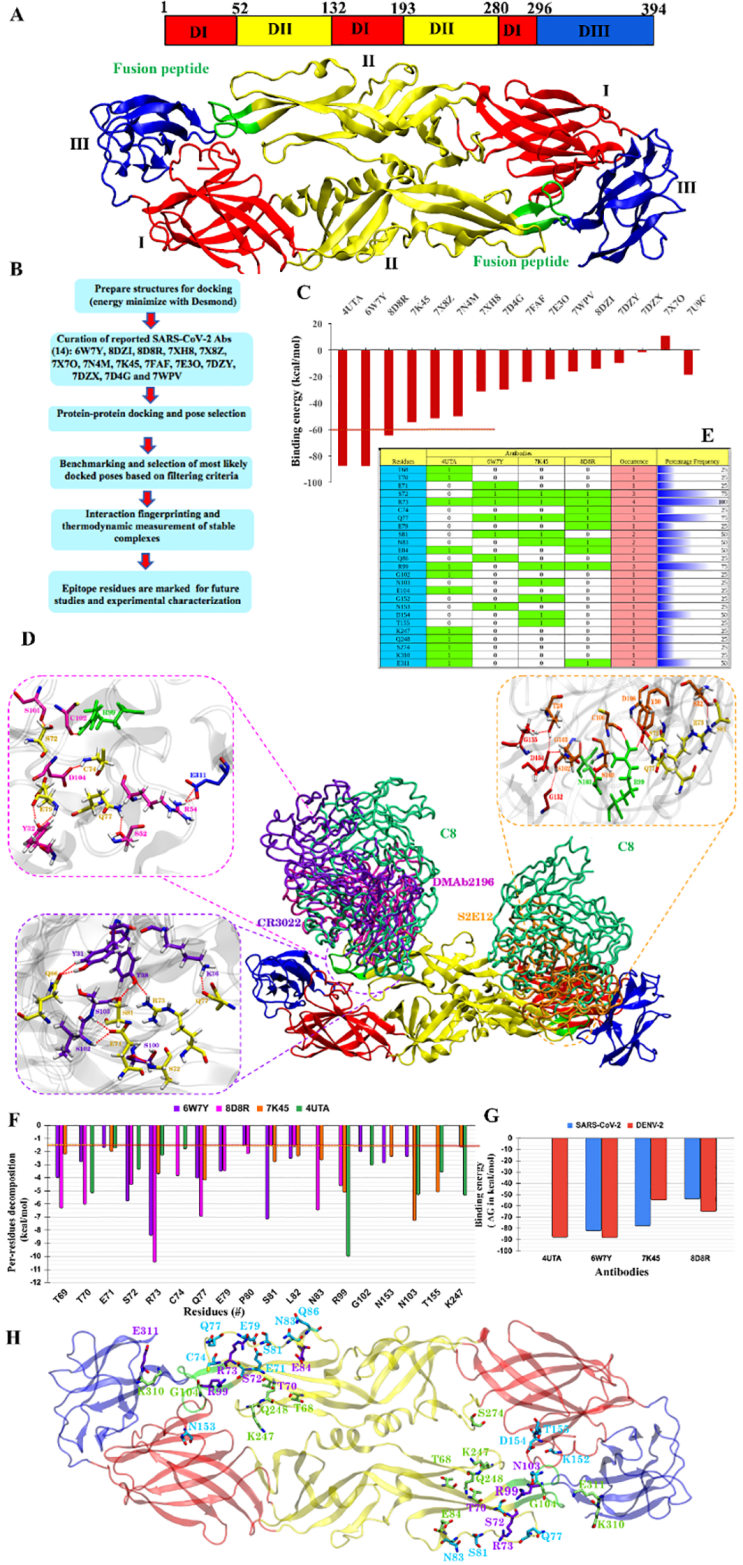
Computational insights exploring the cross-reactive SARS-CoV-2 antibodies at DENV-2 E protein: **(A)** Sequence and structural organization of the DENV-2 E dimer (PDB: 1OAN). **(B)** Overview of computational pipeline. **(C)** Binding free energy (kcal/mol) of selected SARS-CoV-2 antibodies. The most likely complexes were generated using protein–protein docking. **(D)** Interaction map of the top three antibodies (CR3022:6W7Y: violet, S2E12 neutralizing antibody Fab fragment: 7K45: orange, and DMAb 2196:8D8R: magenta) along with the native antibody of DENV-2 E as a control (C8:4UTA: green). The residue-wise zoom-out view is shown to highlight the critical residues mainly forming interactions, such as H bonds and SBs. DENV-2 antibody interactions are shown in green-colored residues, and SARS-CoV-2 antibody interactions are highlighted in pink-colored residues. **(E)** Matrix of critical residues involved in establishing either H bonds or SBs along with their occurrence. **(F)** Per-residue decomposition of the top three antibodies indicating the thermodynamic contributions of epitope residues. **(G)** The net binding energy between the identified antibodies and DENV-2 E, which is claimed to be a potential in both cases. **(H)** 3D positioning of identified epitope residues in the DENV dimer. The residues that only interact with SARS-CoV-2 antibodies are shown in cyan, and those interacting with only DENV-2 antibodies are shown in green. The residues that interact with both DENV-2 E and SARS-CoV-2 RBD are shown in violet. All residues are rendered in licorice and atom-wise C, cyan/green/violet; N, blue; O, red; and S, yellow.

To assess the cross-reactivity of SARS-CoV-2 spike antibodies with DENV-2 E protein dimers, we used an *in silico* approach (**Figures 1A, B**). We selected a panel of reported SARS-CoV-2 antibodies, based on their structural data (extent of their characterization, availability of structural information of their co-crystals with low resolution, preferably below 4 Å) and biological relevance (neutralization potential against SARS-CoV-2, target epitopes, etc.; **Supplementary Table S1**). To identify DENV-2 E epitopes, the C8 antibody against the E protein dimer (Fab region, PDB-id: 4UTA) was used to characterize the interaction site based on interaction fingerprinting. To identify the cross-reactive antibodies forming the most promising complexes with the E-protein, selected SARS-CoV-2 antibodies’ protein–protein (PP) blind docking was performed with the whole DENV-2 E dimer. The consensus-docked poses obtained from the Piper and HDOCK tools were used for post-docking qualitative and quantitative analyses (pose selection and pose-filtering criteria). The top three SARS-CoV-2 antibodies forming complexes with DENV-2 in terms of docking energy, interaction fingerprinting with co-crystal and MM-GBSA scores were CR3022 (Fab region, PDB-id:6W7Y), S2E12 ab (Fab region, PDB-id:7K45), and DMAb 2196 (Fab region, PDB:8D8R) (**Figure 1C**).

To quantify their binding association at the residue level, major contributing amino acids were identified by residue-wise decomposition analysis, setting a cut-off value of −1.5 kcal/mol. For the C8 antibody (4UTA) binding to DENV-2 E protein, the interacting epitopes were majorly localized in Domain II (**Figure 1D**). The binding sites of CR3022, S2E12 ab, and DMAb 2196 antibodies overlapped with the C8 binding site (**Figure 1D**). The contributing residues for all three antibodies were mainly located in Domain II, including the fusion loop (**Figures 1E, F**). From the interaction analysis, R73 appeared to be the most common interacting residue in all antibodies. The pairwise interaction analysis confirmed the considerable overlap in the residues between the SARS-CoV-2 and DENV-2 antibodies (**Figures 1E, F**). Key residues that overlap between DENV-2 and at least two antibodies of SARS-CoV-2 are R73 (common among all), T70, S72 (Domain-II), R99 and N103 (fusion loop) and E311 (Domain-III). We also performed mining of the paratope residues of SARS-CoV-2 antibodies with equal and/or increased interaction energy as C8 and identified overlapping residues between C8 and CR3022 (**Supplementary Figure S1**).

Overall, the interaction maps and per-residue energetic decompositions showed that several common residues were involved in the interaction of DENV-2-E with C8, CR3022, S2E12 ab, and DMAb 2196 antibodies, indicating a high likelihood of cross-reactivity (**Figures 1G, H**, **Supplementary Table S2**). The paratope residues involved in the interaction of the E protein with CR3022 were also identified (**Supplementary Table S3**), and their interactions were assessed (**Supplementary Figure S2**).

### SARS-CoV-2 spike antibody CR3022 cross-reacts with DENV-2 and enhances DENV infection *in vitro*

**Figure 2:**
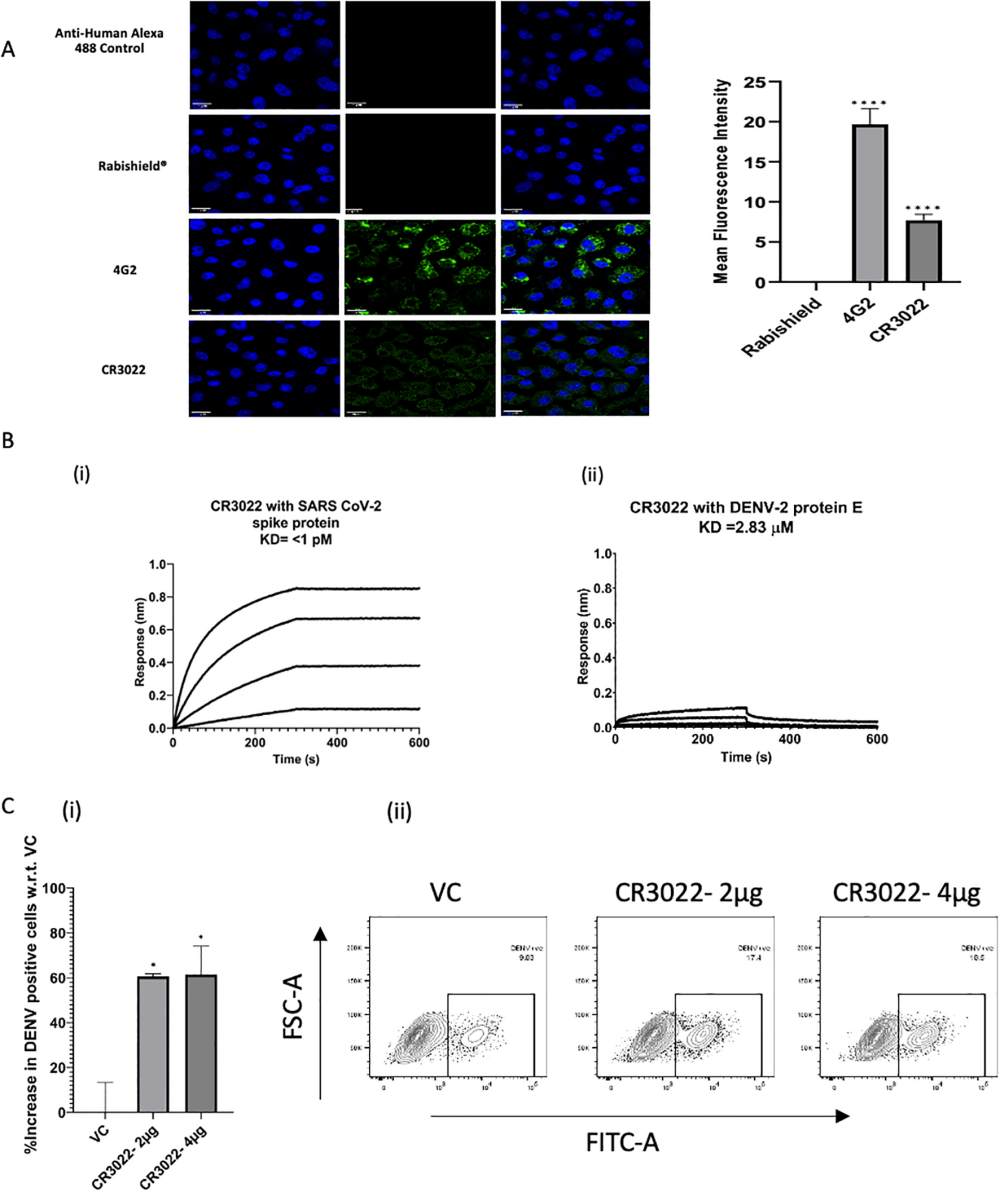
Cross-reactivity and ADE of dengue virus infection by the SARS-CoV-2 anti-RBD antibody CR3022. **(A)** Confocal digital images of DENV-2 (NGC) infected C6/36 cells stained with CR3022 along with the controls, 4G2 and Rabishield^®^ (taken from **Figure 5** to show the comparison) and the Mean Fluorescence Intensity (MFI). **(B)** Binding kinetics of (i) CR3022 with SARS-CoV-2 spike protein and (ii) DENV-2 protein E. **(C)** ADE of DENV-2 infection by different concentrations of CR3022 (2 µg and 4 µg) in K562 cells by flow cytometry. Statistical significance was determined using one-way ANOVA followed by Dunnett’s test. Asterisk (*) indicates a statistically significant difference between the control and treatment. P-value = 0.1234 (ns), 0.0332 (*), 0.0021 (**), 0.0002 (***), <0.0001 (****).

Based on the computational outcomes, one of the antibodies, CR3022, which showed the highest affinity with DENV-2 E protein, was studied further. Based on dengue virus susceptibility and cell monolayer robustness, we selected the C6/36 cell line for confocal studies. Confocal microscopy images of CR3022 showed a statistically significant cross-reactivity with an average Mean Fluorescence Intensity (MFI) of 7.68 (p-value <0.0001) (**Figure 2A**). Bio-Layer Interferometry (BLI) showed a high affinity with a dissociation constant (KD) of <1.0 pM with the spike protein (**Figure 2Bi**) and 2.83 µM with the DENV-2 E protein (**Figure 2Bii**). Furthermore, we observed a significant increase in the percentage of DENV-infected K562 cells with CR3022 antibody (p = 0.01) compared to the untreated virus control (**Figure 2C**).

### SARS-CoV-2 convalescent plasma enhances DENV infection *in vitro*

**Figure 3:**
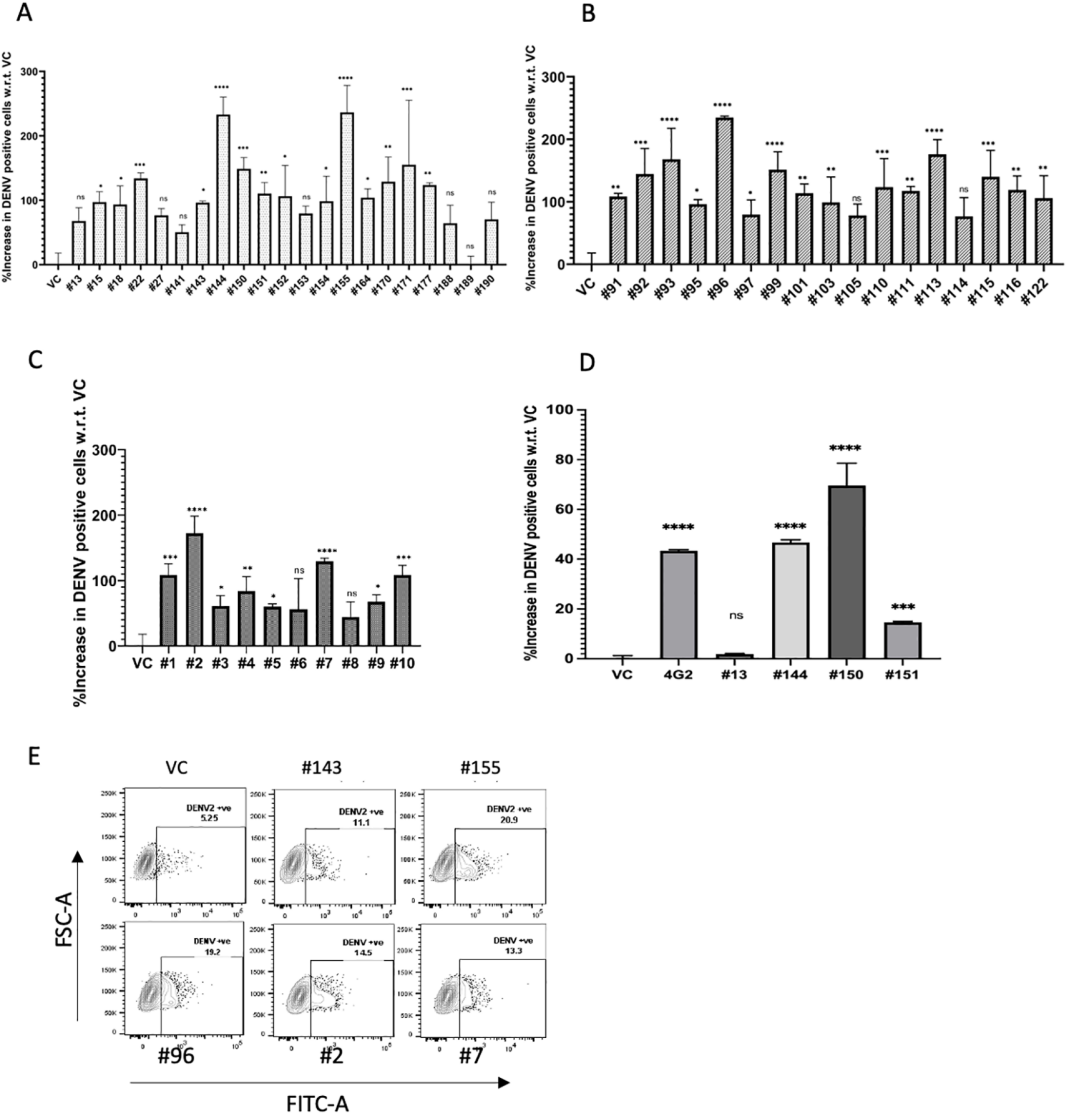
ADE of dengue virus infection by SARS-CoV-2 positive patients’ sera in K562 cells. ADE due to convalescent plasma samples collected in the **(A)** first interval (samples collected from May 2020 to Jan 2021), n = 21, **(B)** second interval (samples collected from May 2021 to June 2021), n = 17, and **(C)** third interval (samples collected from February 2022 to April 2022), n = 10. **(D)** IgG purified (10 µg) from the four convalescent samples showing the strongest ADE activity was assessed for its potential to enhance DENV infection using a dengue clinical strain (IND-60). The bar graph represents the average percentage increase in DENV-positive cells with respect to VC for each sample, with standard deviation for duplicates. Statistical significance was determined using one-way ANOVA followed by Dunnett’s test in GraphPad Prism 8.4.2. Asterisk (*) indicates statistically significant difference between the viral control and serum samples. P-value = 0.1234 (ns), 0.0332 (*), 0.0021 (**), 0.0002 (***), <0.0001 (****). **(E)** Representative dot plots of patient samples from the first interval (#143, #155), second interval (#96), and third interval (#2, #7). Data was analyzed on FlowJo version 10.8.1.

Samples from convalescent COVID-19 patients (n = 48, **Supplementary Table S4**) at different time intervals of the pandemic were tested for their ability to cause ADE. We used FcγR-I, II, and III-expressing K562 and U937 cell lines. Both cell lines predominantly expressed FcγR-II (**Supplementary Figure S3**), as reported previously (23). The basal value of only virus infection obtained in cell lines was normalized to the tested samples to calculate the fold change, and graphs were plotted against virus infection (no serum) vs. virus infection (incubated with serum) in the tested samples. The number of DENV-2 infected K562 cells increased from 1 to 236 times in the first set of convalescent samples (recovered individuals during the Wuhan wave), 77 to 235 times in the second set of convalescent samples (recovered individuals during/around the delta wave), and 44 to 172 times in the third set of convalescent samples (recovered individuals during the omicron wave), compared to the untreated virus control (**Figures 3A–C**). The FACS gating strategy is illustrated in **Supplementary Figure S4**. To confirm that the observed ADE effect was specifically mediated by antibodies present in the serum, IgG was purified from convalescent samples exhibiting high ADE activity. The assay was performed using 10 µg of purified IgG and DENV-2 (clinical strain INDI-60). A significant enhancement of DENV-2 infection was observed in K562 cells incubated with purified IgGs from convalescent samples #144 (P <0.0001), #150 (P <0.0001), and #151 (P <0.0002), compared to the virus control (**Figure 3D**). Representative dot plots of patient samples at different intervals are shown in **Figure 3E**.

Using a subset of the convalescent plasma samples (n = 15), similar results were observed in U937 cells, where DENV-2 infection was enhanced 32–292 times compared to the virus control (**Supplementary Figures S5A, B**). A virus neutralization assay with the SARS-CoV-2 Wuhan strain (**Supplementary Table S5**) did not show any association between the neutralizing antibody titer and ADE in samples collected at different time periods of the SARS-CoV-2 pandemic, as well as with the severity of SARS-CoV-2 infection (**Supplementary Figures S6A–D**).

A representative quantitative PCR assay for intracellular DENV-2 genomic RNA showed a higher viral load in 70% of the COVID-19 convalescent plasma-treated samples than in the control (**Supplementary Figure S7**). This data correlated with the increase in DENV-2 positive cells observed by flow cytometry. The infectious titer of DENV produced from infected cells by focus-forming unit assay also showed a significant increase in the foci-forming units per millimeter (FFU/ml) in COVID-19 convalescent plasma treated samples compared to the control (**Supplementary Figure S8**).

### SARS-CoV-2 antibodies enhance dengue virus infection *in vivo*

**Figure 4:**
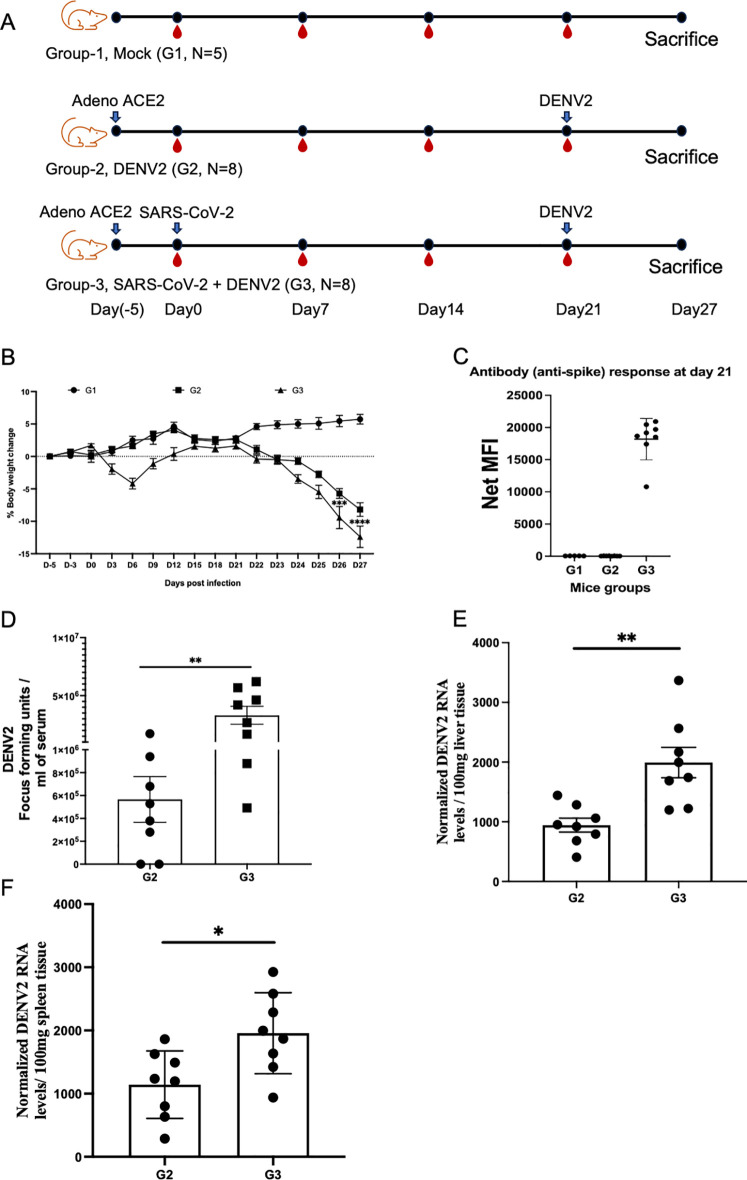
Effect of SARS-CoV-2 on dengue infection in AG129 mice. **(A)** Schematic experimental plan showing three animal groups: Mock as G1, adeno-ACE-2 viral vector infection at 5 days prior to day 0 in G2 and G3, SARS-CoV-2 infection at day 2 followed by infection with dengue virus infection at day 21 in G2, and SARS-CoV-2 infection at day 2 followed by infection with dengue virus at day 21 in G3. **(B)** Change in body weight. Two-way ANOVA was used. **(C)** SARS-CoV-2 spike antibody response on day 21. **(D)** DENV2 viral particles were quantified using the FFU assay in the serum of infected mice. The Mann–Whitney U test was used for statistical analysis to compare the two independent groups. **(E, F)** DENV2 genome was quantified by qRT-PCR from 100 mg of spleen and liver tissue. Based on the small sample size and comparison of the two groups, the Student’s t-test was applied, and the data are represented as mean ± SEM. *P <0.05, **P <0.01, ns, non-significant.

To validate the *in silico* and *in vitro* findings, we performed *in vivo* experiments using AG129 (immunocompromised, lack of IFNAR1 and IFNGR1) mice aged 6–8 weeks (N = 21). The mice were divided into three groups: Group 1 as mock (G1, N = 5), Group 2 as dengue virus control (G2, N = 8), and Group 3 as test with both SARS-CoV-2 and dengue virus infection (G3, N = 8). G2 was infected with dengue virus on day 21, and G3 was infected with SARS-CoV-2 on day 0, followed by dengue virus infection on day 21. Both G2 and G3 mice were infected with the adeno-ACE-2 (Ad5CMVh, The University of Iowa Viral Vector Core Facility) viral vector at −5 days, which is essential for SARS-CoV-2 infection. Body weight was observed for all animals until day 27, when they were euthanized, as shown in the schematic experimental plan (**Figure 4A**). The average body weight of G2 mice decreased on day 3 but recovered over time (**Figure 4B**). A significant decrease in body weight was observed in G3 compared to that in G2.

We further tested the antibody response against SARS-CoV-2 spike antigen on day 21 before DENV-2 infection and observed the anti-spike IgG antibody response in all mice in G3 (**Figure 4C**). Next, the live dengue virus particles were determined in serum samples collected on day 27 (**Figure 4D**). The data revealed a notable increase in the number of viral particles in G3 mice compared to G2 mice, indicating the enhancement of dengue infection with SARS-CoV-2. Next, we tested viral replication in the livers and spleens of infected mice. We observed a high copy number of viral RNA in both the liver (**Figure 4E**) and spleen (**Figure 4F**) tissues. We also observed a decrease in platelet count, as previously reported during dengue infection. As shown in **Supplementary Figure S9A**, a decrease in the platelet count was observed in G1 compared to G2 and G3; however, we did not observe any significant difference between G2 and G3. WBC and hematocrit measurements were also performed but no significant differences were observed between the G2 and G3 groups (**Supplementary Figures S9A, B**).

### SARS-CoV-2 spike antibodies cross-react with DENV-2

**Figure 5:**
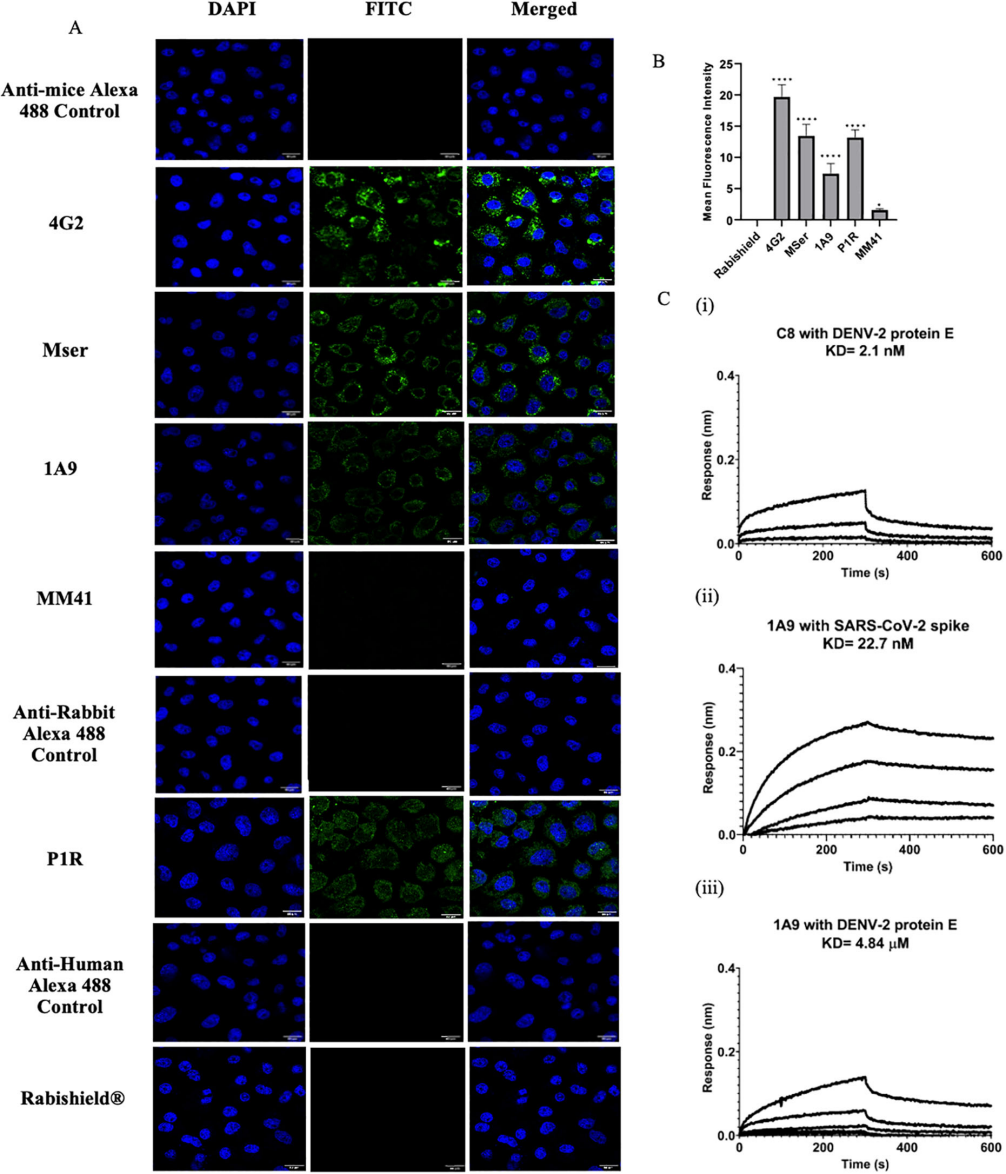
Cross-reactivity of antibodies and sera raised against SARS-CoV-2 spike with DENV-2 (NGC) proteins. **(A)** Confocal digital images of DENV-2 (NGC) infected C6/36 cells reacted with 4G2 antibodies and sera raised against SARS-CoV-2 after 48 hpi. Cell nuclei were stained with DAPI, and the other antibodies were detected using FITC-labeled secondary antibodies. **(B)** Bar graph showing the MFI of 4G2, MSer, 1A9, and P1R (normalized with respect to the respective secondary antibody controls) with standard deviation. Analysis was performed using ImageJ, and statistical significance was determined using one-way ANOVA followed by Dunnett’s test in GraphPad Prism 8.4.2. Asterisk (*) indicates a statistically significant difference in MFI with respect to Rabishield^®^ (negative control). P-value = 0.1234 (ns), 0.0332 (*), 0.0021 (**), 0.0002 (***), <0.0001 (****). **(C)** Binding kinetics of C8 (DENV-E protein specific) and 1A9 (SARS-CoV-2 spike-specific) antibodies with DENV-2 protein E and SARS-CoV-2 spike protein. Antibodies were loaded onto the sensors, and serial dilutions of the proteins were used to study the binding kinetics. The binding responses were calculated by subtracting the data from the reference and fitting globally with a 1:1 binding model using ForteBio’s Data Analysis software 10.1. The data was considered validated if χ2 <0.5.

We wanted to check whether the available sera/antibodies cross-reacted with DENV-2. To do this, SARS-CoV-2 anti-spike polyclonal antibodies raised in rabbits (P1R) and mice (MSer) (obtained from BEI resources, USA) were analyzed for cross-reactivity with DENV-2. Using confocal microscopy, we observed that both showed significant cross-reactivity, with an average (MFI) of 13.2 for P1R and 13.4 for MSer (**Figures 5A, B**). Mouse monoclonal antibodies against SARS-CoV-2, 1A9 (MFI of 7.4) and MM41 (MFI of 1.57), also exhibited cross-reactivity with DENV-2. The positive control, 4G2 (pan-flavivirus-anti-envelope antibody), showed an MFI of 18.28, whereas the negative control, anti-rabies monoclonal (Rabishield^®^), showed no reactivity with DENV-2. The binding kinetics of 1A9 (SARS-CoV-2 spike antibody) showed a high affinity to DENV-2 Envelope with a dissociation constant (KD) of 4.84 µM (**Figures 5Cii–iii**) using Bio-Layer Interferometry (BLI).

### SARS-CoV-2 spike antibodies enhance DENV-2 infection

**Figure 6:**
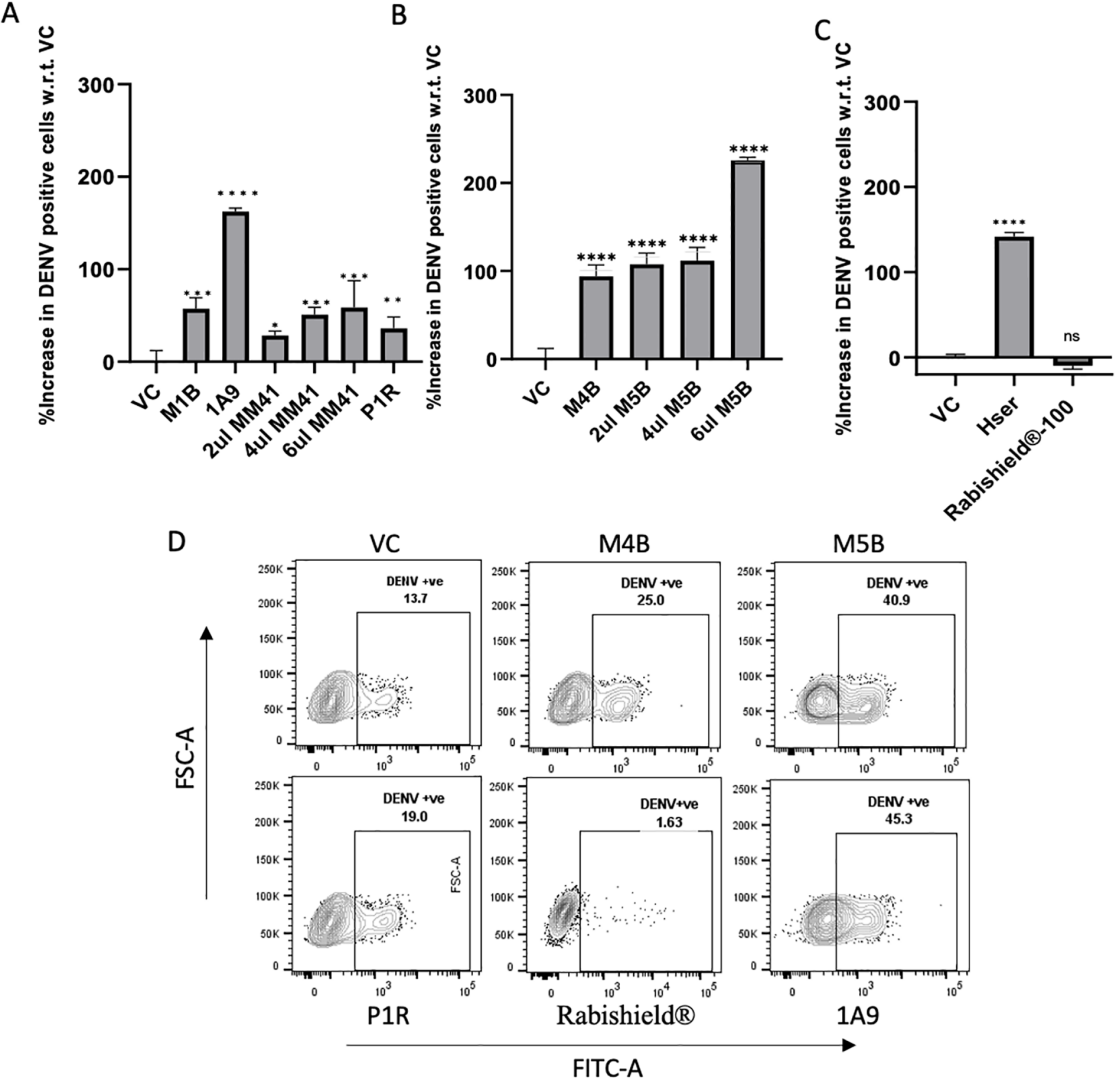
ADE of dengue virus infection by monoclonal and polyclonal antibodies and serum against SARS-CoV-2. ADE due to **(A)** anti-spike monoclonal antibodies, M1B (5 µg), 1A9 (5 µg), and different volumes of MM41 (2 µg, 4 µg, and 6 µg), and anti-spike polyclonal antibody raised in rabbit, P1R (5 µg), **(B)** anti-RBD monoclonal antibodies, M4B (5 µg), and different volumes of M5B (2 µg, 4 µg, and 6 µg), and **(C)** hamster serum immunized with SARS-CoV-2 spike and negative control (Rabishield^®^). Statistical significance was calculated using One-way ANOVA followed by Dunnett’s test in GraphPad Prism 8.4.2. Asterisk (*) indicates statistically significant difference between the viral control and serum samples. P-value = 0.1234 (ns), 0.0332 (*), 0.0021 (**), 0.0002 (***), <0.0001 (****). **(D)** Representative dot plots of the data. Data were analyzed using FlowJo version 10.8.1.

As DENV is endemic to India, pre-existing anti-DENV antibodies may have been responsible for the observed ADE. To rule out this possibility, we further tested commercially available monoclonal and polyclonal antibodies and sera raised in-house against the SARS-CoV-2 spike and RBD for their potential to enhance DENV-2 infection (**Supplementary Table S6**). All tested antibodies (M1B, 1A9, MM41, P1R, M4B, M5B, and Hser) enhanced DENV-2 infection (**Figure 6**). The RBD-specific antibody (M5B) showed stronger enhancement than the antibody against spike (MM41), with a dose-dependent increase in DENV-2 infection (**Figures 6A, B**). Similarly, hamster serum against purified SARS-CoV-2 spike protein also showed ADE of DENV-2 infection in K562 cells (**Figure 6C**). Monoclonal antibodies against the rabies virus (negative control) did not show any ADE of dengue infection in K562 cells (**Figure 6C**). A similar ADE of DENV infection was observed in U937 cells (**Supplementary Figure S10**). Three monoclonal antibodies—4G2, 1A9, and M4B—showed significant ADE, as determined by the infectious titer of the virus released in the culture supernatant from K562 cells (p-value <0.0001; **Supplementary Figure S10**).

Having established that there is an enhancement of DENV-2 infection due to anti-SARS-CoV-2 antibodies, we next investigated whether this was time-dependent. We assessed ADE at 6 h, 18 h and 36 h post-infection (**Supplementary Figure S11**). Mice (Mser) and hamster (Hser) sera showed significant ADE with respect to virus control (adjusted p-values Mser = 0.0071 and Hser = 0.0001), even at 6 hpi (**Supplementary Figure S10**). However, we did not observe a significant enhancement due to 4G2 and anti-spike monoclonals M3S and M2G at 6 hpi. At 18 hpi, a significant increase in DENV-2 positive cells with respect to the virus control was observed in 4G2 as well as M2G (adjusted p-value = 0.0101), which was further significantly enhanced at 36 hpi (adjusted p-values 4G2 = 0.0001 and M2G = 0.0035). The ADE in Hser reached saturation as early as 6 h (adjusted p-value <0.0001).

## Discussion

In this study, we demonstrated that SARS-CoV-2 antibodies cross-react with DENV-2 and enhance DENV-2 infection. Our *in silico* analysis indicated a high likelihood of cross-reactivity between SARS-CoV-2 spike antibodies and the DENV-2 E protein. The spike protein is critical for viral entry into cells and is the main target of both neutralizing and infection-enhancing antibodies. Docking results showed strong interactions of SARS-CoV-2 antibodies with the DENV-2 E dimer, with binding free energies for CR3022 (6W7Y), S2E12 (7K45), and DMAb2196 (8D8R) comparable to the DENV-2-specific antibody C8 (4UTA). These three SARS-CoV-2 antibodies shared an interacting interface with C8, and residues contacted by C8 on DENV-2 E were also contacted by at least one of these antibodies and contributed the most to stabilizing their binding. We also found that convalescent plasma from SARS-CoV-2–recovered individuals, collected at three different time points during the COVID-19 pandemic, significantly enhanced DENV-2 infection in Fc receptor-positive K562 and U937 cells.

Although these cell lines are widely used as a model to study ADE, we acknowledge that they cannot fully recapitulate the complexity of primary immune cells. We observed an increased viral load, as indicated by FFU, and increased viral RNA levels in the spleen and liver of AG129 mice that had SARS-CoV-2 antibodies. This *in vivo* study provides additional evidence that SARS-CoV-2 antibodies can enhance dengue infection in a physiological context and overcomes the limitations of using *in vitro* model systems. We also plan to use primary human monocytes/macrophages to assess ADE under physiologically relevant conditions.

Our results support the findings of Reinig et al. (24), who showed that SARS-CoV-2 vaccination generates cross-reactive antibodies against DENV with a distinct IgM- and IgA-biased isotype profile. Importantly, even in the presence of low anti-dengue IgG titers, these antibodies were sufficient to trigger ADE *in vitro*. Our work also supports the published data by others, highlighting SARS-CoV-2 cross-reactivity with the dengue virus and false positivity with dengue serology (25, 26). We used the DENV-2 envelope protein for BLI and computational studies because it has been reported to have maximum cross-reactivity with SARS-CoV-2 S1 specific antibodies (20). The dimeric DENV-2 E-protein was used for docking studies as we intended to study the interaction of DENV-2 with SARS-CoV-2 spike antibodies, which would happen before the viral entry, extracellularly and it is known that the E protein exists as a head-to-tail oriented homodimer on the surface of mature virions (27). Mapping of all residues contacted by the three strongest SARS-CoV-2 binders showed that most lie in domain II and the fusion loop of E. Antibodies against two DENV epitopes are known as strong mediators of ADE: prM epitopes (28) and fusion loop epitopes (29), suggesting that fusion loop epitopes are likely involved in ADE in our system. *In silico* interaction mining revealed several critical residues including R73 (common to all three antibodies), T70 and S72 (domain II), R99 and N103 (fusion loop), and E311 (domain III), highlighting potential sites for future epitope-based therapeutic interventions.

The DENV-2 used in our *in vitro* assays was grown in C6/36 cells and was not formally characterized for the ratio of immature to mature virions. Viruses produced in mosquito cells typically contain more immature particles than those produced in mammalian cells, likely because of differences in furin expression (30, 31). Laboratory-adapted strains are more dynamic than clinical isolates and readily expose cryptic epitopes (32). Thus, our preparation likely contained a high proportion of immature virions, but this should not prevent ADE, as natural DENV infections also involve heterogeneous virus populations. In natural infection, the antibody repertoire is enriched for poorly neutralizing antibodies generated mainly against immature conformations (33), which arise from incomplete furin processing and lead to the release of both immature and mature virions after assembly (34, 35). Also, the fully mature virions have been reported to be less susceptible to neutralization by cross-reactive and heterotypic immune sera than partially mature variants (33). Thus, the heterogeneity in the DENV-2 preparation used for *in vitro* assays is likely closely related to the natural infection scenario. Due to the lack of information about pre-exposure to DENV in our patient samples, which may influence the results, we used commercially available SARS-CoV-2 antibodies and spike-immunized animal sera to prove that the ADE effect is due to SARS-CoV-2 antibodies. Although we only tested ADE against DENV serotype 2 (NGC strain and clinical isolate (Indi-60) strain), we expect to observe an ADE effect with a differential degree of ADE with DENV serotypes 1, 3, and 4, since they are antigenically related.

However, in the animal experiment, the platelet, WBC count, and hematocrit levels were not significantly different between the mice given dengue (G2) and those infected with SARS-CoV-2 + DENV (G3). As these parameters are associated with disease severity, this could explain why we did not observe dengue severity during the SARS-CoV-2 pandemic, which are promotes us to dig deeper and conduct further studies to find the missing links. We also observed an increase in dengue viral load in G3 (SARS-CoV-2 + DENV-infected) group mice compared to G2 (dengue-infected) group mice, which might indicate an increase in the number of symptomatic cases in the endemic population rather than asymptomatic cases. It is still unclear whether there was underreporting or reduced testing for dengue during the peak SARS-CoV-2 pandemic, as the clinical symptoms of both viruses are very similar. Our understanding remains limited, and future studies may shed light on this issue.

The limitation of the study is the availability of pre-pandemic clinical samples, such as plasma/serum or PBMCs, which could have provided better insight and might have shed some light on why these factors do not lead to dengue severity in the real-world scenario. We attempted to overcome this using an animal model, but a clinical sample would have been a better comparator.

Based on the current findings from *in silico*, *in vitro*, and *in vivo* experiments, the study suggests that SARS-CoV-2 spike-directed antibodies cross-react with the dengue envelope protein and enhance the dengue viral load in AG129 experimental mice. Our findings highlight the importance of cross-reactivity and viral interactions in endemic regions where more than one virus circulates simultaneously. Understanding viral interactions during the co-circulation/co-infections requires further research to understand how they pose vaccine development challenges and impact public health. These factors will also pose significant challenges to surveillance and/or vaccine running programs, which might provide false positivity due to viral cross-reactivity. Therefore, an assay that can clearly differentiate viral infections will be a more beneficial approach in the future.

## Materials and methods

### Molecular docking of DENV-2 envelope glycoprotein and SARS-CoV-2 antibodies

#### Curation of data

The X-ray crystal structures of the DENV-2 envelope glycoprotein (PDB ID:4UT6) (36) and SARS-CoV-2 antibodies were curated from the RCSB in the PDB format (http://www.rcsb.org/pdb). These crystal structures, along with their corresponding resolution values, are summarized in **Supplementary Table S1**. The crystal structures of the antibodies were curated by removing spike proteins, water molecules, and metal ions using UCSF Chimera 1.15 (37). All structures were prepared using Maestro’s Protein Preparation Wizard module (Schrödinger Release 2020-1) (38). Hydrogens and bond orders were added, and missing side chains and loops were filled using Prime (38). Hydrogen bond (HB) optimization and restrained minimization were performed for the systems using force field OPLS4 (39).

#### Protein–antibody docking and choosing the best pose

Protein–protein interactions govern various aspects of structural and functional cellular mechanisms, and their elucidation is crucial for understanding of biological processes. All the prepared SARS-CoV-2 antibodies, along with controls, that is, positive (pdb-id:4UTA) and negative control (pdb-id:7U9G), were docked on the prepared DENV2 E protein structure by masking the non-CDR regions of the antibodies. The Chothia definition was used for the CDR region. Multiple docking methods were used to minimize bias, including HDOCK (40), pyDock (41), and Piper (42). The rationale for implementing multiple docking programs was to obtain consensus results from all three tools and remove any false-positive outcomes. The best poses were chosen from the docked models based on their docking score, cluster size (43), and reported paratope residues (44, 45).

#### Thermodynamic quantification using molecular mechanics-generalized born surface area

The binding free energy of the protein and antibody complexes was calculated using the molecular mechanics generalized born surface area (MM-GBSA) method in the Prime (46) module of the Schrodinger suite. We used the VSGB solvation model and the OPLS4 force field. The binding free energy is calculated by: MMGBSA dG Bind (NS) = Complex − Receptor (from optimized complex) − Ligand (from optimized complex) = MMGBSA dG Bind − Rec Strain − Lig Strain, where, Rec Strain = Receptor (from optimized complex) − Receptor, Lig Strain = Ligand (from optimized complex) − Ligand, MMGBSA dG Bind = Complex − Receptor − Ligand, NS, no strain.

#### Figures

All figures were generated using VMD (V.1.9.1) (47) and graphs were generated using XMGRACE (Version 5.1.19).

#### Cells

Human myeloid cell lines K562 (ATCC CCL-243) and U937 (ATCC CRL-1593.2) were cultured in Iscove’s Modified Dulbecco’s media (IMDM, HIMEDIA) and Roswell Park Memorial Institute (RPMI, Gibco) 1640, respectively. African Green Monkey cell line Vero E6 (NCCS, India) was maintained in Dulbecco’s Modified Eagle Medium (DMEM, HIMEDIA) while *A. albopictus* cell line C6/36 (NCCS, India) was cultivated in Leibovitz’s L-15 Medium (HIMEDIA). All cells were maintained in the respective media supplemented with 100 U/ml of penicillin, 100 μg/ml streptomycin (HIMEDIA), and 10% fetal bovine serum (HIMEDIA). C6/36 cells were maintained at 28°C (ESCO incubator) without CO_2_, whereas the other cells were grown in a humidified 37 °C incubator (Thermo Fisher Scientific) with 5% CO_2_.

#### Virus stocks

Dengue virus serotype 2 (NGC strain) and (P8-P23085 INDI-60) were propagated in C6/36 cells and used for all assays. Cells at a confluency of 60%–70% were infected with dengue virus at a multiplicity of infection (MOI) of 0.1 using an inoculum prepared in L-15 medium supplemented with 2% FBS. The cells were incubated with the antibody for 2 h at 28°C with intermittent rocking. Subsequently, the cells were washed and incubated with L-15 medium containing 10% FBS. Culture supernatant was harvested at 5 dpi, clarified by centrifugation at 129×*g* at room temperature and concentrated using centrifugal ultrafiltration (Amicon^®^ Ultra centrifugal filter (100 kDa), ACS510024 from Millipore, Darmstadt, Germany) at 3,220×*g* for 10 min for every 15 ml of supernatant. The infectious titer of the virus was determined using a focus-forming unit (FFU) assay performed on Vero E6 cells.

The three variants of SARS-CoV-2, original Wuhan (USA-WA1/2020, GenBank: MN985325), B.1.617.2 (δ-variant) known as THSTI_287 (GenBank: MZ356566.1), and Omicron BA.1 (GISAID accession no.: EPI_ISL_8764350) were propagated in Vero E6 cells. Cells were infected with a 0.1 MOI of the viral inoculum and incubated for 1 h at 37°C with shaking. The viral supernatant was harvested at 48 hpi, filtered, and stored at −80°C. The infective titer was determined by titration of Vero E6 cells using the TCID_50_ method (48). These viral stocks were used for neutralization assays.

#### Serum samples and antibodies

All human clinical samples used in this study were obtained from the THSTI Biorepository after approval by the Institutional Ethics Committee on Biomedical and Health Research [THS 1.8.1/ (196)]. We selected a group of convalescent plasma samples (n = 48) collected at three different time intervals during the COVID-19 pandemic in India. They are defined as follows: the first interval was from May 2020 to January 2021, the second from May 2021 to June 2021, and the third from February 2022 to April 2022. The clinical details of the samples are presented in **Supplementary Table S1**. The pan flavivirus, anti-envelope mAb, 4G2 was purified in-house from HB112 hybridoma cells. SARS-CoV-2 spike (S2 specific) antibody 1A9 was purchased from Gentex, and anti-recombinant SARS-CoV-2 spike protein (whole spike) Sino 4059-MM41 was purchased from Sino Biologicals. Other SARS-CoV-2-related antibodies M1B, M4B, M5B, and P1R were obtained from BEI Resources, USA. SARS-CoV-2 infected mice and hamster serum were generated in-house. Rabies Human monoclonal antibody, Rabishield^®^-100 (Serum Institute of India), was purchased from a pharmacy. All details regarding the commercial antibodies/serum are mentioned in **Supplementary Table S2**.

#### Confocal microscopy

Approximately 0.5 × 10^6^ C6/36 cells from an exponentially growing culture were seeded on glass cover slips, followed by infection with DENV-2 at an MOI of 1.0. At 48 h post-infection, the cells were fixed with 3.7% paraformaldehyde (PFA) and permeabilized using 0.1% Triton-X-100. The cells were immunostained with primary and fluor-conjugated secondary antibodies. Subsequently, the coverslips were mounted onto glass slides using ProLong Gold Antifade Mountant supplemented with DAPI. Fluorescence was observed at ×60 magnification using a FLUOVIEW FV3000 confocal microscope (Olympus). The mean fluorescence intensity (MFI) was calculated using images from 10 fields per slide, with seven cells per field (at least 70 data points). Statistical analysis was performed using one-way ANOVA followed by Dunnett’s test, comparing the negative control (Rabishield).

#### Binding kinetics using biolayer interferometry

BLI studies were performed using an Octet RED 96 instrument at 24 °C with shaking at 1,000 rpm (ForteBio). For the DENV-2 Envelope protein, the buffer composition was 50 mM Tris, 150 mM NaCl, 0.5% glycerol, pH 8.0. For the SARS-CoV-2 spike protein, phosphate-buffered saline (pH 7.4) was used. The sensors were placed in the respective kinetic buffer (49). To study the binding kinetics of protein E, antibodies at a concentration of 5 µg/ml were loaded onto the sensors (AHC (Anti-hIgG Fc Capture) for CR3022 and C8, protein G (for 1A9 antibody)). The DENV E dimeric protein was serially diluted three-fold (starting with 6,000 nM), and the spike trimeric protein was diluted 2-fold (starting with 600 nM) in their respective kinetic buffers. The designed experiment started with a baseline (100 s), followed by three cycles of neutralization and regeneration, and a baseline (100 s). After this step, antibody loading was performed (700 s) on all five sensors. After the loading step, another baseline (baseline2, 60 s), was established, and association was performed after the 2nd baseline step for 300 s, followed by 300 s of dissociation.

Here the 5th sensor was used as a reference, where no analyte was used, and the analyte concentration was maintained at 0 nM. The same sensor during the data processing was assigned as the reference well (change well type-reference well), and the same wells (reference well) were subtracted from the raw data. Here, the ligand and analyte dilutions, as well as the entire experiment were performed in the respective kinetic buffers. The binding responses were calculated and fitted globally with a 1:1 binding model using ForteBio’s Data Analysis software (version 10.1). The data were considered validated if X^2^ <0.5.

#### Antibody-dependent enhancement assay

Convalescent serum samples (15 μl), purified IgG antibodies (10 µg), and commercial antibodies (at various concentrations) were diluted in plain Iscove’s Modified Dulbecco’s Medium (IMDM) to a total volume of 100 μl. Subsequently, 100 μl of viral dilution was added, and the mixture was incubated at 37°C for 1 h. The virus–antibody complexes were then allowed to infect 1 × 10^6^ K562/U937 cells for 2 h at an MOI of 1. After infection, the cells were washed and incubated in IMDM-2% FBS at 37°C, 5% CO_2_ for 36 h. Subsequently, the cells were fixed using 4% PFA, permeabilized with 0.1% Triton X-100, immunostained with purified 4G2 (dilution 1:200), and Alexa-Fluor 488 conjugated anti-mouse (dilution 1:500). The number of cells positive for the DENV antigen was quantified using flow cytometry.

### Isolation and purification of IgG from serum samples

Serum samples were centrifuged at 10,000×*g* at 4°C for 10 min, followed by the addition of one part of 1 M Tris–HCl, pH 8.0 to 10 parts of serum sample, to adjust the pH. Saturated ammonium sulfate solution (up to 45% saturation) was added dropwise to the solution, and the mixture was stirred for 1 h. The serum sample was centrifuged again at 10,000×*g* at 4°C for 10 min, and the supernatant was discarded. The pellet was dissolved in a minimum volume of the desalting column buffer. The Sephadex G-25 column was cleaned with distilled water and then equilibrated with 10 ml–15 ml of binding buffer. The sample was loaded onto the column, and the flow-through was collected in a fresh 15 ml Falcon tube. The binding buffer (5 ml–10 ml) was passed through the column again, and approximately 1 ml–2 ml of the flow-through was collected in the same Falcon tube. To separate IgG, the protein G column was rinsed with ethanol and distilled water. Collection tubes with 50 μl of 1.0 M dibasic sodium phosphate were prepared to ensure that the final pH of the collected samples was close to neutral. The packed column was washed with five column volumes (approximately 25 ml) of distilled water to remove the slurry storage medium. The column was equilibrated with five to 10 column volumes of 50 mM sodium phosphate, pH 7.0, and 500 mM NaCl (the binding buffer). The pre-treated sample was loaded onto an equilibrated column. The column was washed with approximately 25 ml binding buffer, followed by elution with five column volumes of elution buffer. Fractions of 500 μl were collected in collection tubes containing neutralizing buffer. The fractions were analyzed by absorbance at 280 nm in a spectrophotometer to measure protein concentration and SDS-PAGE to confirm the purity of IgG.

### Flow cytometry analysis

Flow cytometry was performed using a BD FACS Canto II flow cytometer (BD Biosciences) with 20,000 cells acquired per sample using FACS Diva software. FlowJo v10.8.1 was used for the analysis. Live cells were gated based on FSC and SSC, followed by singlet determination using the FSC-A vs. FSC-H gate. Dengue virus-positive cells were gated in the FITC channel using a singlet population. The FITC voltage was set using the isotype control. Fcγ receptor expression was assessed using anti-CD64 (Alexa Fluor 700), anti-CD32 (PE), and anti-CD16 (APC) antibodies in K562 and U937 cells. Statistical analyses were performed using GraphPad Prism 8.

### Live virus neutralization assay

Two-fold serial dilutions of heat-inactivated plasma samples were mixed with 100 TCID_50_ of SARS-CoV-2 and subsequently allowed to infect Vero E6 monolayers. After incubation for 72 h at 37°C with 5% CO_2_, the degree of CPE was qualitatively determined using a microscope. The absence of CPE indicated virus neutralization, and the highest dilution that completely prevented the emergence of CPE was considered the neutralization titer. For plasma samples collected during the third interval, cells were fixed 24 h post-infection and immunostained using an antibody against the SARS-CoV-2 nucleocapsid protein (1:2,000, 4H2, Genscript), followed by Alexa 488 conjugated anti-mouse antibody (1:500, Invitrogen). Fluorescent foci were quantified using the AID iSpot Reader with AID EliSpot 8.0 iSpot software.

### Detection of DENV RNA using quantitative RT-PCR

Total RNA was extracted from 1 × 10^6^ K562 cells using the standard TRIzol method. RNA was quantified using Nanodrop (Thermo Fisher Scientific), and 300 ng RNA was used for qPCR using a one-step RT-PCR kit (SOLIScript^®^ 1-step Probe kit, Solis Biodyne) following the manufacturer’s protocol for the detection of DENV2 RNA. GAPDH was used as an internal control. The fold change in DENV2 RNA copies was calculated with respect to GAPDH. The details of the primer and probe sequences are provided in **Supplementary Table S7**.

### Focus forming unit assay

FFU assay was carried out to determine the virus titer in the serum of DENV2 infected AG129 mice with slight modifications. Vero E6 cells were seeded at a density of 0.01 × 10^6^ cells/well in a 96-well black/clear bottom plate, and the cells were allowed to reach 80% confluency. At 80% confluency, 100 µl of 10-fold serially diluted serum in media with 2% FBS was added to the wells and incubated. After 2 h, the serum was removed, fresh media with 2% FBS was added, and the cells were incubated for 24 h at 37°C in 5% CO_2_. Cells were fixed with 2% PFA, and immunostaining for virus detection was performed using mAb 4G2 (pan-DENV anti-envelope antibody), followed by the secondary antibody, goat anti-mouse IgG, AF488 conjugated (Invitrogen) prepared in permeabilization buffer (0.1% Triton ×100 with 2%BSA in PBS). Foci were visualized and counted using a fluorescence microscope.

### Mouse infection

AG129 (IFN-α/β/γR −/− 129/Sv) mice were obtained from the Jackson Laboratory (Bar Harbor, ME, USA) and maintained in a small animal facility (SAF) at the RCB, Faridabad, India. All experiments were performed using AG129 mice aged 6–8 weeks, of either sex. One group of mice was treated (one-time through the intranasal route) with a human ACE2 adenovirus using 2.5 × 10^8^ PFU for transient expression of hACE2 in the lungs and respiratory tract. After five days, the mice were infected with SARS-CoV-2 via nasal route inoculation using 1 × 10^5^ PFU of the virus. Mice were bled on day 7, 14, and 21 post SARS-CoV-2 infection to measure antibodies against SARS-CoV-2. At day 21, both groups were challenged with 1 × 10^4^ FFU mouse-adapted DENV2 (P8-P23085 INDI-60) and were euthanized in a moribund state. Mice were anesthetized using 4% isoflurane (inhalation) to collect blood via the retro-orbital sinus. Euthanasia was performed after anesthetizing the mice by injecting them with ketamine (100 mg/kg) and xylazine (10 mg/kg) in a CO_2_ chamber. The method of euthanasia adopted was the CO_2_ asphyxiation technique, strictly following the recommendations of the American Veterinary Medical Association (AVMA) guidelines, 2020. A CO_2_ chamber was used, and the flow rate of CO_2_ was maintained at 2 l/min with a gradual displacement of 30% of the chamber volume. Flow was maintained for 5 min to ensure proper euthanasia of the animal.

### Quantitative real time PCR

Total RNA was extracted from mice tissues using RNAiso (Takara Bio, Japan), followed by phenol-chloroform treatment. First-strand cDNA was synthesized from 1 µg RNA using a cDNA synthesis kit (BioRad, USA) according to the manufacturer’s protocol. cDNA was used for real-time PCR using SYBR Green Supermix (BioRad) in an Applied Biosystems Quant Studio™ 6 Flex Real-Time PCR System. The primer sets used for the detection of the respective genes are listed in **Supplementary Table S8**.

### Statistical analysis

Experimental values are represented as mean ± standard deviation (SD). Statistical analysis was performed using ordinary one-way ANOVA followed by Dunnett’s multiple comparison test using GraphPad Prism version 8.4.2. Differences were considered significant at p <0.05.

## Data Availability

The original contributions presented in the study are included in the article/**Supplementary Material**. Further inquiries can be directed to the corresponding author.

## References

[1] WHO . WHO, Fact sheets / Detail / Dengue and severe dengue 2023. Available online at: https://www.who.int/news-room/fact-sheets/detail/dengue-and-severe-dengue (Accessed March 23, 2025).

[2] Lee MF Voon GZ Lim HX Chua ML Poh CL . Innate and adaptive immune evasion by dengue virus. Front Cell Infect Microbiol. (2022) 12:1004608. doi: 10.3389/fcimb.2022.1004608, PMID: 36189361 PMC9523788

[3] Pierson TC Diamond MS . A game of numbers: the stoichiometry of antibody-mediated neutralization of flavivirus infection. Prog Mol Biol Transl Sci. (2015) 129:141–66. doi: 10.1016/bs.pmbts.2014.10.005, PMID: 25595803 PMC4910618

[4] Sharif N Opu RR Saha T Masud AI Naim J Alsharif KF . Evolving epidemiology, clinical features, and genotyping of dengue outbreaks in Bangladesh, 2000-2024: a systematic review. Front Microbiol. (2024) 15:1481418. doi: 10.3389/fmicb.2024.1481418, PMID: 39539699 PMC11557403

[5] Rodriguez DM Madewell ZJ Torres JM Rivera A Wong JM Santiago GA . Epidemiology of dengue - Puerto Rico, 2010-2024. MMWR Morb Mortal Wkly Rep. (2024) 73:1112–7. doi: 10.15585/mmwr.mm7349a1, PMID: 39666586 PMC11637419

[6] Hernandez Bautista PF Cabrera Gaytan DA Santacruz Tinoco CE Vallejos Paras A Alvarado Yaah JE Martinez Miguel B . Retrospective analysis of severe dengue by dengue virus serotypes in a population with social security, Mexico 2023. Viruses. (2024) 16:769. doi: 10.3390/v16050769, PMID: 38793650 PMC11125731

[7] Narvaez F Montenegro C Juarez JG Zambrana JV Gonzalez K Videa E . Dengue severity by serotype and immune status in 19 years of pediatric clinical studies in Nicaragua. medRxiv. (2024). doi: 10.1101/2024.02.11.24302393, PMID: 39792951 PMC11750095

[8] Alagarasu K Patil JA Kakade MB More AM Yogesh B Newase P . Serotype and genotype diversity of dengue viruses circulating in India: a multi-centre retrospective study involving the Virus Research Diagnostic Laboratory Network in 2018. Int J Infect Dis. (2021) 111:242–52. doi: 10.1016/j.ijid.2021.08.045, PMID: 34428547

[9] Khan S Akbar SMF Yahiro T Mahtab MA Kimitsuki K Hashimoto T . Dengue infections during COVID-19 period: reflection of reality or elusive data due to effect of pandemic. Int J Environ Res Public Health. (2022) 19:10768. doi: 10.3390/ijerph191710768, PMID: 36078486 PMC9518125

[10] Khan S Akbar SMF Nishizono A . Co-existence of a pandemic (SARS-CoV-2) and an epidemic (Dengue virus) at some focal points in Southeast Asia: Pathogenic importance, preparedness, and strategy of tackling. Lancet Reg Health Southeast Asia. (2022) 4:100046. doi: 10.1016/j.lansea.2022.100046, PMID: 35873345 PMC9296506

[11] Lu X Bambrick H Pongsumpun P Dhewantara PW Toan DTT Hu W . Dengue outbreaks in the COVID-19 era: Alarm raised for Asia. PloS Negl Trop Dis. (2021) 15:e0009778. doi: 10.1371/journal.pntd.0009778, PMID: 34624031 PMC8500420

[12] Manna S Satapathy P Bora I Padhi BK . Dengue outbreaks in South Asia amid Covid-19: Epidemiology, transmission, and mitigation strategies. Front Public Health. (2022) 10:1060043. doi: 10.3389/fpubh.2022.1060043, PMID: 36589966 PMC9797821

[13] Oladipo HJ Rabiu I Tajudeen YA . Dengue virus and SARS-CoV-2 Co-infection dynamics: An emerging threat among African countries. Ann Med Surg (Lond). (2022) 82:104398. doi: 10.1016/j.amsu.2022.104398, PMID: 36035770 PMC9394095

[14] Prapty C Rahmat R Araf Y Shounak SK Noor AA Rahaman TI . SARS-CoV-2 and dengue virus co-infection: Epidemiology, pathogenesis, diagnosis, treatment, and management. Rev Med Virol. (2023) 33:e2340. doi: 10.1002/rmv.2340, PMID: 35238422 PMC9111128

[15] Rana MS Usman M Alam MM Ikram A Salman M Umair M . Impact of previous SARS-CoV-2 infection on the rate of mortality in dengue. A preliminary report from Pakistan. J Infect. (2022) 84:722–46. doi: 10.1016/j.jinf.2022.01.027, PMID: 35081438 PMC8783977

[16] Masyeni S Santoso MS Widyaningsih PD Asmara DW Nainu F Harapan H . Serological cross-reaction and coinfection of dengue and COVID-19 in Asia: Experience from Indonesia. Int J Infect Dis. (2021) 102:152–4. doi: 10.1016/j.ijid.2020.10.043, PMID: 33115680 PMC7585717

[17] Alla D Alla SSM Vempati R Bhatt H Sultana Q Bhatt S . Dengue & COVID-19: A comparison and the challenges at hand. Cureus. (2022) 14:e31877. doi: 10.7759/cureus.31877, PMID: 36579259 PMC9792364

[18] Digwo DC Elebe CP Chigor VN Madueke SN Ezeh CK Ike AC . Occurrence of false-positive tests and cross-reactions between COVID-19 and dengue with implications during diagnosis: A mixed evidence synthesis. Infect Microbes Diseases. (2023) 5:64–75. doi: 10.1097/im9.0000000000000116

[19] Ulrich H Pillat MM Tarnok A . Dengue fever, COVID-19 (SARS-coV-2), and antibody-dependent enhancement (ADE): A perspective. Cytometry A. (2020) 97:662–7. doi: 10.1002/cyto.a.24047, PMID: 32506725 PMC7300451

[20] Cheng YL Chao CH Lai YC Hsieh KH Wang JR Wan SW . Antibodies against the SARS-CoV-2 S1-RBD cross-react with dengue virus and hinder dengue pathogenesis. Front Immunol. (2022) 13:941923. doi: 10.3389/fimmu.2022.941923, PMID: 36045680 PMC9420930

[21] Nath H Mallick A Roy S Kayal T Ranjan S Sengupta S . COVID-19 serum can be cross-reactive and neutralizing against the dengue virus, as observed by the dengue virus neutralization test. Int J Infect Dis. (2022) 122:576–84. doi: 10.1016/j.ijid.2022.07.013, PMID: 35811081 PMC9262656

[22] El-Qushayri AE Kamel AMA Reda A Ghozy S . Does dengue and COVID-19 co-infection have worse outcomes? A systematic review of current evidence. Rev Med Virol. (2022) 32:e2339. doi: 10.1002/rmv.2339, PMID: 35213764 PMC9111070

[23] Littaua R Kurane I Ennis FA . Human IgG Fc receptor II mediates antibody-dependent enhancement of dengue virus infection. J Immunol. (1990) 144:3183–6. doi: 10.4049/jimmunol.144.8.3183, PMID: 2139079

[24] Reinig S KC Huang S-Y Hsiung K-C Chen P-K Ito E Lee I-K . COVID-19 vaccination induces cross-reactive dengue virus antibodies with altered isotype profiles and *in vitro* antibody-dependent enhancement. Front Immunol. (2025) 16:1683070. doi: 10.3389/fimmu.2025.1683070, PMID: 41479919 PMC12753992

[25] Yan G Lee CK Lam LTM Yan B Chua YX Lim AYN . Covert COVID-19 and false-positive dengue serology in Singapore. Lancet Infect Dis. (2020) 20:536. doi: 10.1016/S1473-3099(20)30158-4, PMID: 32145189 PMC7128937

[26] Lustig Y Keler S Kolodny R Ben-Tal N Atias-Varon D Shlush E . Potential antigenic cross-reactivity between severe acute respiratory syndrome coronavirus 2 (SARS-coV-2) and dengue viruses. Clin Infect Dis. (2021) 73:e2444–e9. doi: 10.1093/cid/ciaa1207, PMID: 32797228 PMC7454334

[27] Modis Y Ogata S Clements D Harrison SC . A ligand-binding pocket in the dengue virus envelope glycoprotein. Proc Natl Acad Sci U.S.A. (2003) 100:6986–91. doi: 10.1073/pnas.0832193100, PMID: 12759475 PMC165817

[28] Rodenhuis-Zybert IA van der Schaar HM da Silva Voorham JM van der Ende-Metselaar H Lei HY Wilschut J . Immature dengue virus: a veiled pathogen? PloS Pathog. (2010) 6:e1000718. doi: 10.1371/journal.ppat.1000718, PMID: 20062797 PMC2798752

[29] de Alwis R Williams KL Schmid MA Lai CY Patel B Smith SA . Dengue viruses are enhanced by distinct populations of serotype cross-reactive antibodies in human immune sera. PloS Pathog. (2014) 10:e1004386. doi: 10.1371/journal.ppat.1004386, PMID: 25275316 PMC4183589

[30] Murray JM Aaskov JG Wright PJ . Processing of the dengue virus type 2 proteins prM and C-prM. J Gen Virol. (1993) 74:175–82. doi: 10.1099/0022-1317-74-2-175, PMID: 8429301

[31] Davis CW Nguyen HY Hanna SL Sanchez MD Doms RW Pierson TC . West Nile virus discriminates between DC-SIGN and DC-SIGNR for cellular attachment and infection. J Virol. (2006) 80:1290–301. doi: 10.1128/JVI.80.3.1290-1301.2006, PMID: 16415006 PMC1346927

[32] Chaichana P Okabayashi T Puiprom O Sasayama M Sasaki T Yamashita A . Low levels of antibody-dependent enhancement *in vitro* using viruses and plasma from dengue patients. PloS One. (2014) 9:e92173. doi: 10.1371/journal.pone.0092173, PMID: 24642752 PMC3958444

[33] Beltramello M Williams KL Simmons CP Macagno A Simonelli L Quyen NT . The human immune response to Dengue virus is dominated by highly cross-reactive antibodies endowed with neutralizing and enhancing activity. Cell Host Microbe. (2010) 8:271–83. doi: 10.1016/j.chom.2010.08.007, PMID: 20833378 PMC3884547

[34] Pierson TC Diamond MS . Degrees of maturity: the complex structure and biology of flaviviruses. Curr Opin Virol. (2012) 2:168–75. doi: 10.1016/j.coviro.2012.02.011, PMID: 22445964 PMC3715965

[35] Cherrier MV Kaufmann B Nybakken GE Lok SM Warren JT Chen BR . Structural basis for the preferential recognition of immature flaviviruses by a fusion-loop antibody. EMBO J. (2009) 28:3269–76. doi: 10.1038/emboj.2009.245, PMID: 19713934 PMC2771083

[36] Rouvinski A Guardado-Calvo P Barba-Spaeth G Duquerroy S Vaney MC Kikuti CM . Recognition determinants of broadly neutralizing human antibodies against dengue viruses. Nature. (2015) 520:109–13. doi: 10.1038/nature14130, PMID: 25581790

[37] Pettersen EF Goddard TD Huang CC Couch GS Greenblatt DM Meng EC . UCSF Chimera–a visualization system for exploratory research and analysis. J Comput Chem. (2004) 25:1605–12. doi: 10.1002/jcc.20084, PMID: 15264254

[38] Sastry GM Adzhigirey M Day T Annabhimoju R Sherman W . Protein and ligand preparation: parameters, protocols, and influence on virtual screening enrichments. J Comput Aided Mol Des. (2013) 27:221–34. doi: 10.1007/s10822-013-9644-8, PMID: 23579614

[39] Lu C Wu C Ghoreishi D Chen W Wang L Damm W . OPLS4: improving force field accuracy on challenging regimes of chemical space. J Chem Theory Comput. (2021) 17:4291–300. doi: 10.1021/acs.jctc.1c00302, PMID: 34096718

[40] Yan Y Tao H He J Huang SY . The HDOCK server for integrated protein-protein docking. Nat Protoc. (2020) 15:1829–52. doi: 10.1038/s41596-020-0312-x, PMID: 32269383

[41] Pallara C Jimenez-Garcia B Romero M Moal IH Fernandez-Recio J . pyDock scoring for the new modeling challenges in docking: Protein-peptide, homo-multimers, and domain-domain interactions. Proteins. (2017) 85:487–96. doi: 10.1002/prot.25184, PMID: 27701776

[42] Kozakov D Brenke R Comeau SR Vajda S . PIPER: an FFT-based protein docking program with pairwise potentials. Proteins. (2006) 65:392–406. doi: 10.1002/prot.21117, PMID: 16933295

[43] Lorenzen S Zhang Y . Identification of near-native structures by clustering protein docking conformations. Proteins. (2007) 68:187–94. doi: 10.1002/prot.21442, PMID: 17397057

[44] Zhang L Cao L Gao XS Zheng BY Deng YQ Li JX . A proof of concept for neutralizing antibody-guided vaccine design against SARS-CoV-2. Natl Sci Rev. (2021) 8:nwab053. doi: 10.1093/nsr/nwab053, PMID: 34676098 PMC8083607

[45] Shan S Mok CK Zhang S Lan J Li J Yang Z . A potent and protective human neutralizing antibody against SARS-coV-2 variants. Front Immunol. (2021) 12:766821. doi: 10.3389/fimmu.2021.766821, PMID: 34966387 PMC8710476

[46] Wang E Sun H Wang J Wang Z Liu H Zhang JZH . End-point binding free energy calculation with MM/PBSA and MM/GBSA: strategies and applications in drug design. Chem Rev. (2019) 119:9478–508. doi: 10.1021/acs.chemrev.9b00055, PMID: 31244000

[47] Humphrey W Dalke A Schulten K . VMD: visual molecular dynamics. J Mol Graph. (1996) 14:33–8, 27–8. doi: 10.1016/0263-7855(96)00018-5, PMID: 8744570

[48] Reed LJ Muench H . A simple method of estimating fifty per cent endpoints12. Am J Epidemiol. (1938) 27:493–7. doi: 10.1093/oxfordjournals.aje.a118408

[49] Zhang M Wang H Foster ER Nikolov ZL Fernando SD King MD . Binding behavior of spike protein and receptor binding domain of the SARS-CoV-2 virus at different environmental conditions. Sci Rep. (2022) 12:789. doi: 10.1038/s41598-021-04673-y, PMID: 35039570 PMC8763896

