# Supplementary material for: SARS-CoV-2 spike antibodies cross-react with dengue virus and enhance infection in vitro and in vivo
Source: Front Immunol. 2026 Jan 15;16:1724625. doi: 10.3389/fimmu.2025.1724625 (PMC12852381; doi:10.3389/fimmu.2025.1724625)
Supplement: Supplementary file 1 [file DataSheet1.pdf]

Supplementary Data

Appendix

Supplementary Table S1: Details of antibodies for computational study -------------------04

Supplementary Table S2: Details of common epitopes indicated by computational study08

Supplementary Table S3: Details of paratope residues involved in interaction of E-protein with CR3022--09

Supplementary Table S4: Clinical data of the 48 patient samples taken for study--------10

Supplementary Table S5: Neutralizing antibody titers of the commercial SARS-CoV-2 antibodies, animal sera, and convalescent plasma samples against the original Wuhan strain--11

Supplementary Table S6: Details of antibodies and sera used for the study---------------15

Supplementary Table S7: Primer and probe sequences used of DENV and GAPDH in qRT PCR---17

Supplementary Table S8: Primer and probe sequences used in qRT PCR for animal tissues---18

Supplementary Figure S1: Mining of antibodies interacting residues-----------------------19

Supplementary Figure S2: Protein interaction diagram showing paratope residues of mAb CR3022 (chain H L) involved in interaction with E-protein of SARS-CoV-2 (Chain A B).---20

Supplementary Figure S3: Expression of Fcγ Receptors (FcγRs), type I, II, and III in undifferentiated K562 and U937 cells---21

Supplementary Figure S4: Gating strategy for detection of DENV-positive cells in flowcytometry---22

Supplementary Figure S5: ADE of dengue virus infection by the SARS-CoV-2 positive patients' samples in U937 cells--23

Supplementary Figure S6: Comparison of neutralizing antibody titres of patient samples against SARS-CoV-2 Wuhan with the increase in dengue virus infection--------24

Supplementary Figure S7: Quantitation of dengue virus ADE using qRT PCR-----------25

Supplementary Figure S8: Replicating virus in supernatants from ADE of dengue by the antibodies and convalescent plasma samples against SARS-CoV-2 in K562 cells---------26

Supplementary Figure S9. Haematological analysis of dengue-infected AG129 mice following SARS-CoV-2 challenge. ---**27**

Supplementary Figure S9: ADE of dengue virus infection by the monoclonal and polyclonal antibodies and serum against SARS-CoV-2 in U937 cells-------------------28

Supplementary Figure S10: Time point-based kinetics of ADE of dengue virus infection by the SARS-CoV-2 anti-Spike monoclonal antibodies and sera raised in mice and hamsters against Spike--29

References:--- 30

Supplementary Table S1: Details of antibodies for computational study

Sr. No.	Antibody	PDB-ID	Resolution (Å)	Neutralization status	kD	target	References
1	Fab14 antibody	7WPV	2·46	Neutralizing	4·14nM	RBD	[1]
2	Fab fragment of FC05 antibody	7D4G	3·90	Neutralizing	4·322 nM	NTD	[2]
3	Fab fragment of 8D2 antibody	7DZX	3·53	Non-Neutralizing (Infection enhancing Ab)	NA	NTD	[3]
4	Fab fragment of 2490 antibody	7DZY	3·60	Non-Neutralizing (Infection enhancing Ab)	NA	NTD	[3]
5	nCoV617 antibody	7E3O	2·51	Neutralizing	4·23 nM	RBD	[4]

6	P36-5D2 antibody	7FAF	3·69	Broadly neutralizing	6·91 nM	RBD	[5]
7	Fab fragment of S2E12 antibody	7K45	3·70	Neutralizing	1·6 nM	RBD	[6]
8	WRAIR-2151 antibody	7N4M	3·79	Neutralizing	<1 pM	RBD	[7]
9	UT28K antibody	7X7O	3·75	Broadly neutralizing	NA	RBD	[8]
10	Fab fragment of Ab188 antibody	7X8Z	4·10	Neutralizing	NA	RBD	[9]
11	Fab fragment of ZCB11 antibody	7XH8	2·99	Neutralizing	57·5 pM	RBD	[10]
12	DMAb 2196 antibody	8D8R	4·10	Neutralizing	NA	RBD	[11]

13	Fab fragment (MB.02) of CoV-0213 antibody	8DZI	3·50	Broadly neutralizing	3·27nM (BA.1)	Omicron RBD	12
14	CR3022 antibody	6W7Y	3·30	Neutralizing	115nM (Fab) <0·1 nM (IgG)	RBD	13
15	Rabies virus antibody RVA122	7U9G	3·39	Neutralizing (Rabies virus)	NA	RABV-G	14
16	Fab fragment of C8 antibody	4UTA	3·00	Broadly neutralizing (DENV)	9 nM	DENV-E	15
17	SARS-CoV-2 RBD in complex	6W41	3·08	Neutralizing	<0·1nM	RBD	13

	with human antibody CR3022						

Supplementary Table S2: Details of common epitopes indicated by computational study

	Antibodies			
	DENV-2	SARS-CoV-2		
Residues	C8:4UTA	CR3022:6W7Y	S2E12:7K45	DMAb 2196:8D8R
R73	1	1	1	1
T70	1	1	0	1
S72	1	1	0	1
R99	1	0	1	1
N103	1	1	1	0

Supplementary Table S3: Details of paratope residues involved in interaction of E-protein with CR3022

DENV2 E protein	CR3022	Domain of E protein
B:Q86	L:Y31	Domain II
B:S81	L:Y38	Domain II
B:Q77	L:K36	Domain II
B:R73	L:Y38	Domain II
B:S72	H:S100	Domain II
B:E71	H:I102 , HS103	Domain II
A:N153	H:Y27	Domain I

Supplementary Table S4: Clinical data of the 48 patient samples taken for the study.

**Clinical factors**	**Total 48**
First interval	21
Second interval	17
Third interval	10
**Gender**	
Male	27 (56·25%)
Female	21 (43·75%)
**Age (Mean)**	41 yrs
Below 18 yrs	1
18-35 yrs	18
36-60 yrs	23
61 above	6
**Symptoms**	
Symptomatic	40
Asymptomatic	8
**Severity**	
ICU admission	6
Oxygen required	11
Ventilation required	1
**Vaccination status**	
Only 1 dose	9
Both doses	35
Non vaccinated	4

Supplementary Table S5: Neutralizing antibody titers of the commercial SARS-CoV-2 antibodies, animal sera, and convalescent plasma samples against the original Wuhan strain, obtained from live virus micro-neutralization assay. For the antibodies, sera and plasma samples in the first and second intervals, reciprocal of the highest dilution at which no cytopathic effect is observed is taken as nAb titre. For samples collected in the third interval, $FRNT_{50}$ values are reported.

Antibodies and Serum samples		
**ID**	**SNT titer against Wuhan**	**Remarks**
4G2	10	Negative
M1B	10	Negative
P1R	10	Negative
M4B	10	Negative
MM41	40	Positive
1A9	10	Negative
Mser	320	Positive
Hser	320	Positive
Rabishield®-100	10	Negative
**First interval**		
**ID**	**SNT titer against Wuhan**	**Remarks**
#13	1280	Positive
#15	640	Positive
#18	160	Positive
#22	2560	Positive

#27	320	Positive
#141	1280	Positive
#143	20	Negative
#144	160	Positive
#150	2560	Positive
#151	1280	Positive
#152	160	Positive
#153	40	Positive
#154	640	Positive
#155	2560	Positive
#164	40	Positive
#170	160	Positive
#171	320	Positive
#177	640	Positive
#188	2560	Positive
#189	160	Positive
#190	2560	Positive
**Second interval**		
**ID**	**SNT titer against Wuhan**	**Remarks**
#91	320	Positive
#92	10	Negative
#93	40	Positive
#95	10	Negative

#96	160	Positive
#97	640	Positive
#99	20	Negative
#101	80	Positive
#103	40	Positive
#105	20	Negative
#110	10	Negative
#111	10	Negative
#113	160	Positive
#114	80	Positive
#115	10	Negative
#116	20	Negative
#122	80	Positive
**Third interval**		
ID	SNT titer against Wuhan	Remarks
#1	545·55	Positive
#2	482·63	Positive
#3	258·87	Positive
#4	248·2	Positive
#5	66·05	Positive
#6	195·08	Positive
#7	246·06	Positive
#8	453·72	Positive

#9	3752·35	Positive
#10	124·44	Positive

Supplementary Table S6: Details of antibodies and sera used for the study

Sr. No.	Antibody/Serum	Details	Source
1	1A9	SARS-CoV / SARS-CoV-2 (COVID-19) spike antibody (S2 subunit), Mouse Mab	Genetex (Cat. No. GTX632604)
2	MM41	SARS-CoV-2 Spike Antibody, Mouse MAb	SinoBiological (Cat. No. 40591-MM41)
3	CR3022	SARS-CoV-2 Spike Protein (CR3022) Human IgG1 mAb (RBD specific)	Cell Signalling Technology (Cat. No. 37475)
4	M1B	NR-56488 Monoclonal Anti-SARS-Related Coronavirus 2 Spike Glycoprotein, Clone 1-3D7 (produced in vitro)	BEI resources
5	M4B	NR-55296 Monoclonal Anti-SARS-Related Coronavirus 2 Spike Glycoprotein Receptor Binding Domain (RBD), Clone 2TP1C3 (produced in vitro)	BEI resources

6	M5B	NR-55295<br>Monoclonal Anti-SARS-Related Coronavirus 2 Spike Glycoprotein Receptor Binding Domain (RBD), Clone 2TP1B11 (produced in vitro)	BEI resources
7	P1R	NR-52947<br>Polyclonal Anti-SARS-Related Coronavirus 2 Spike Glycoprotein (IgG, Rabbit)	BEI resources
8	Rabishield®-100	Rabies Human Monoclonal Antibody (rDNA) (100IU)<br>(By Serum Institute of India Ltd.)	Purchased from Pharmacy
9	4G2	Pan flavivirus anti envelope antibody, Mouse MAb	Inhouse purified from Hb112 hybridoma cells
10	MSer	Mice serum immunized with SARS-CoV-2 Spike	Inhouse
11	HSer	Hamster serum immunized with SARS-CoV-2 Spike	Inhouse

Supplementary Table S7: Primer and probe sequences used of DENV and GAPDH in qRT PCR

Gene		Sequence
**GAPDH**	Forward primer	5'- ATTCCACCCATGGCAAATTC- 3'
	Reverse primer	5'- CGCTCCTGGAAGATGGTGAT- 3'
	Probe	5' -(FAM)- AGCTTCCCGTTCTCAGCCTTCAC -(BHQ1)- 3'
**DENV**	Forward primer	5’- GGTTAGAGGAGACCCCTCCC -3’
	Reverse primer	5’- GGCGTTCTGTGCCTGGA -3’
	Probe	5’- JOE-CAGGATCTCTGGTCTCTCCCAGCGT–BHQ1 -3’

Supplementary Table S8: Primer and probe sequences used in qRT PCR for animal tissues

Gene	Forward Primer (5'-3')	Reverse Primer (5'-3')
Mouse GAPDH	ACCACAGTCCATGCCATCAC	TCCACCACCCTGTTGCTGTA
DENV-2	GCAGAAACACAACATGGAACGATAGT	TGATGTAGCTGTCTCCGAATGG

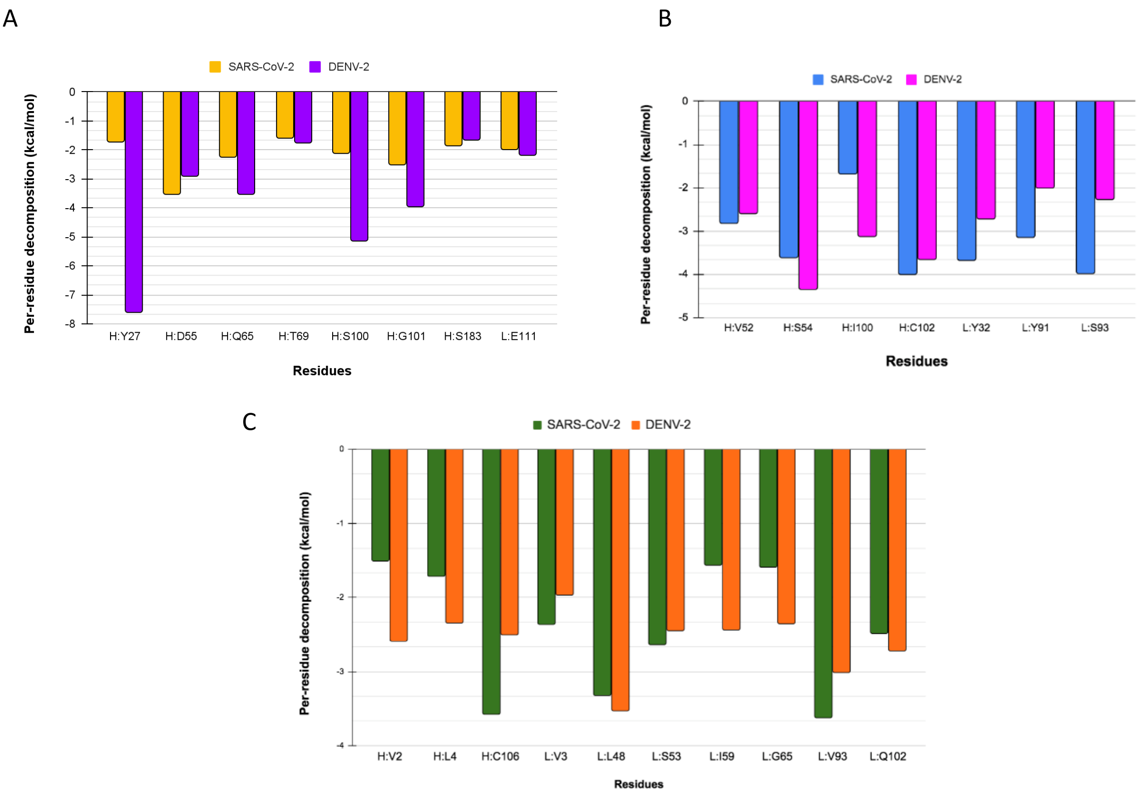

Supplementary Figure S1: Mining of antibodies interacting residues: Paratope residues involve interaction with SARS-CoV-2 and DENV-2. (A) CR3022, (B) DMAb 2196, and (C) S2E12. The color code remains the same.

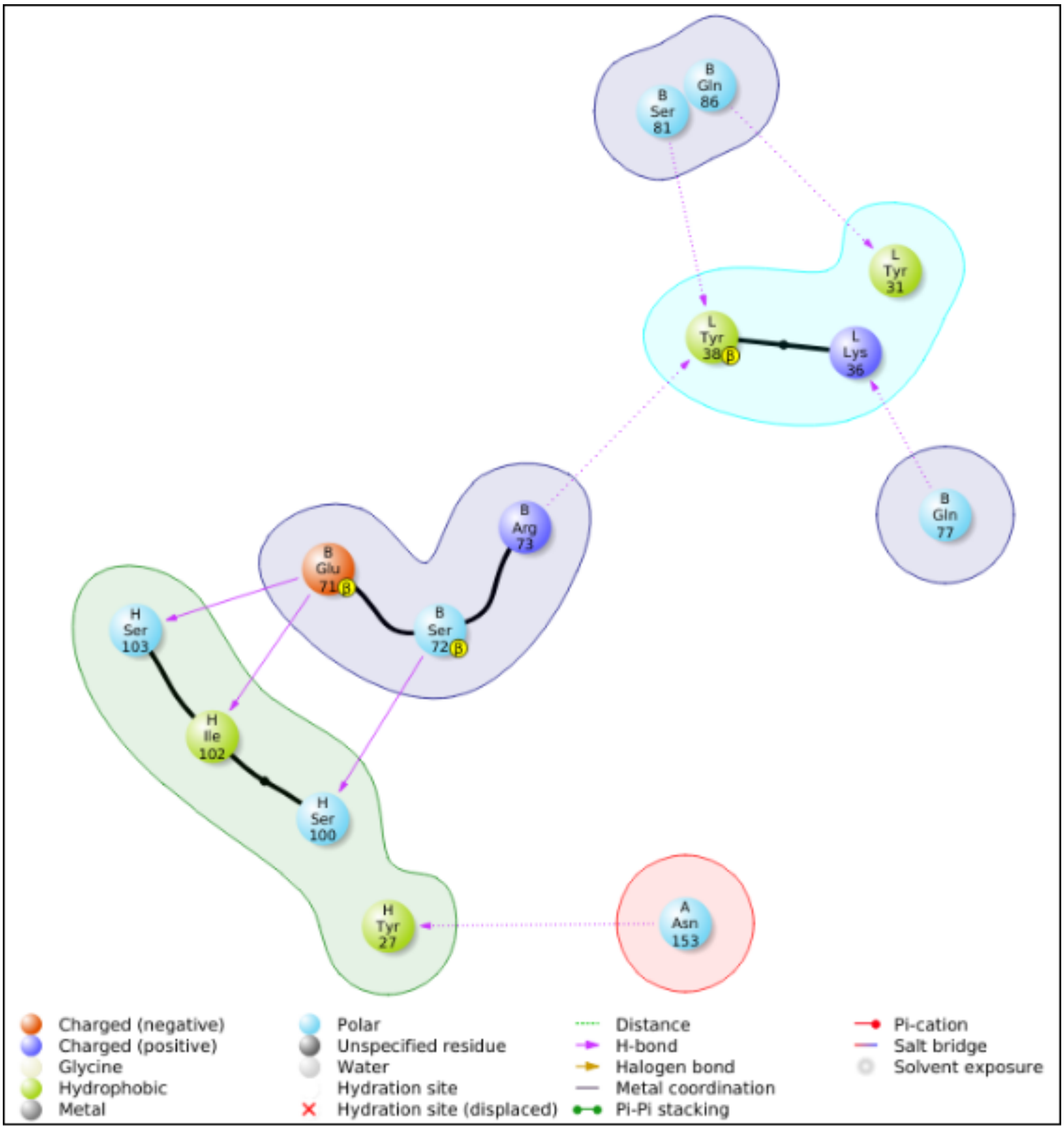

Supplementary Figure S2: Protein interaction diagram showing paratope residues of mAb CR3022 (chain H L) involved in interaction with E-protein of SARS-CoV-2 (Chain A B).

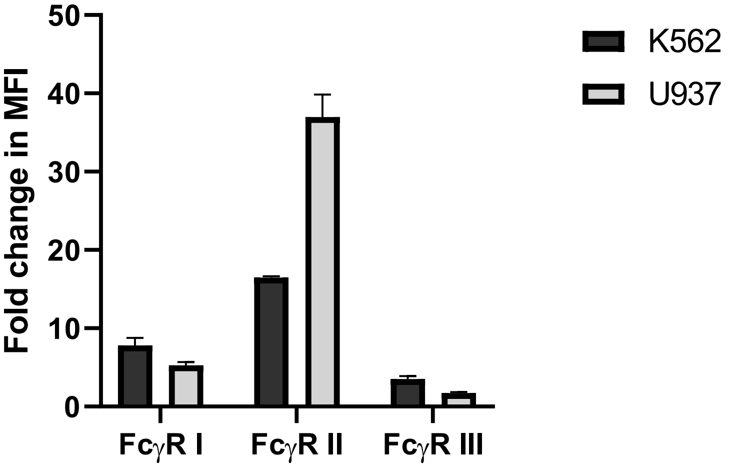

Supplementary Figure S3: Expression of Fcγ Receptors (FcγRs), type I, II and III in undifferentiated K562 and U937 cells. K562 and U937 cells were stained with Alexa flour 700 conjugated anti-CD64, PE conjugated anti-CD32, and APC conjugated anti-CD16 antibodies, for assessing the expression of FcγRI, FcγRII, and FcγRIII respectively. The fold change in MFI was calculated with respect to the unstained cells and plotted as the average MFI with standard deviation.

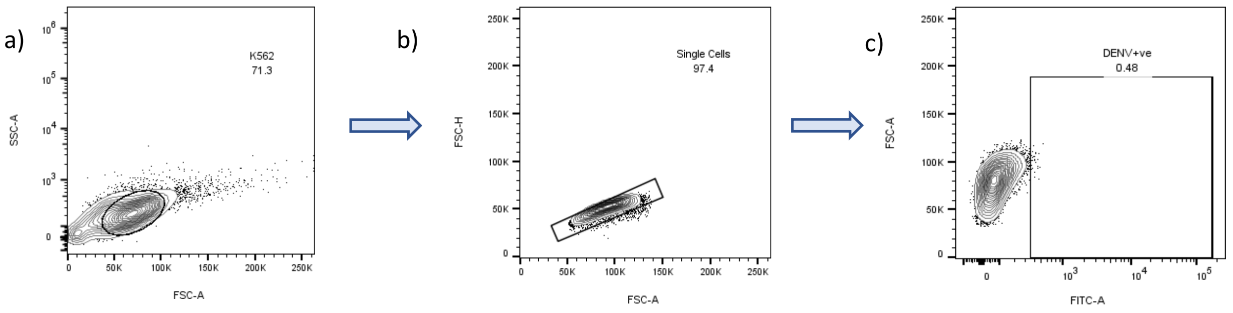

Supplementary Figure S4: Gating strategy for detection of DENV positive cells in flowcytometry.

Flowcytometry was used for the detection of Dengue virus-positive cells using pan flavivirus envelope antibody, 4G2 followed by anti-mouse Alexa 488 secondary antibody.

a) Unstained cells were used to set the gates for live population using FSC-A vs SSC-A. b) singlet cells were selected from live population using FSC-A vs FSC-H. c) The singlet cells were used for gating Dengue virus-positive cells using FITC-A vs FSC-A and the percentage of dengue virus positive cells was recorded for further calculations. FITC voltage was set using an isotype control antibody. Data analysis was done on FlowJo v. 10·8·1 and statistical analysis was performed using GraphPad Prism 8·4·2.

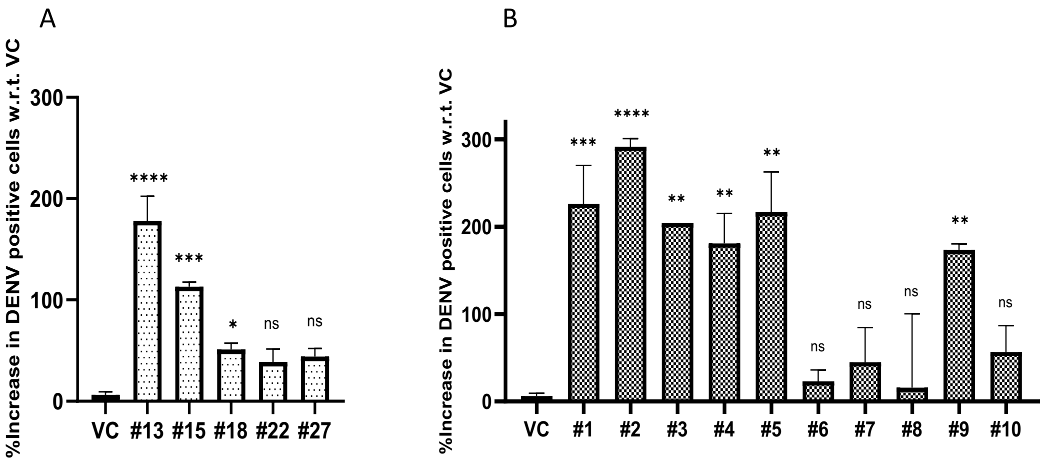

Supplementary figure S5: ADE of dengue virus infection by the SARS-CoV-2 positive patients' samples in U937 cells. ADE due to convalescent plasma samples collected in the (A) first interval, n=5 (B) third interval, n=10. Statistical significance was calculated using One-way ANOVA followed by Dunnett's test. Asterisk (*) indicates that the difference between virus control and serum samples is statistically significant. P value = 0·1234 (ns), 0·0332 (*), 0·0021 (**), 0·0002 (***), <0·0001 (****).

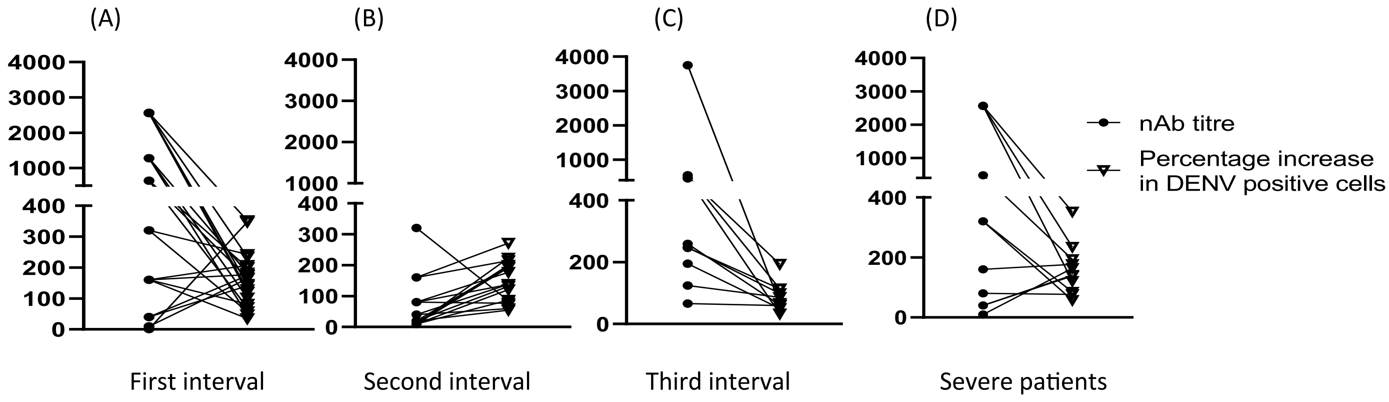

Supplementary Figure S6: Comparison of neutralizing antibody titres of patient samples against SARS-CoV-2 Wuhan with the increase in dengue virus infection. nAb titres of patients against SARS-CoV-2 Wuhan were compared to the ability to cause ADE in terms of percentage increase in dengue virus-positive cells in flowcytometry. The comparison was made with samples collected during (A) first interval, n=21 (B)second interval, n=17, (C) third interval, n=10, and (D) Severe patients. The neutralization titres are taken as the reciprocal of the highest dilution showing CPE for samples in first and second intervals. nAb of negative samples (<1: 20) was considered to be 10 Data was analyzed on GraphPad Prism 9·4·1.

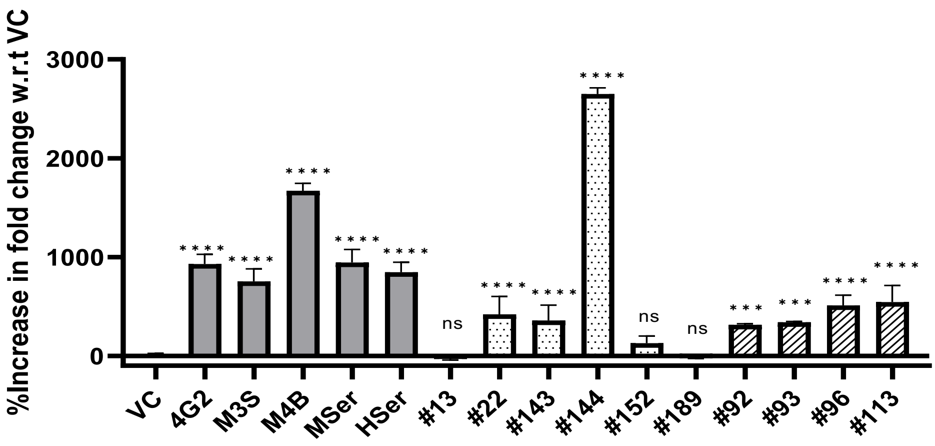

Supplementary Figure S7: Quantitation of dengue virus ADE using qRT PCR. ADE assay was performed followed by quantitative real time PCR for detection of DENV2 RNA and the percentage increase in the fold change of DENV-2 RNA copies was measured with respect to the virus control. GAPDH was used as an internal control. Statistical significance was carried out using One-way ANOVA followed by Dunnett's test. Asterisk (*) indicates that difference between the virus control and the serum samples is statistically significant ($p < 0·0001$). P value = 0·1234 (ns), 0·0332 (*), 0·0021 (**), 0·0002 (***), <0·0001 (****).

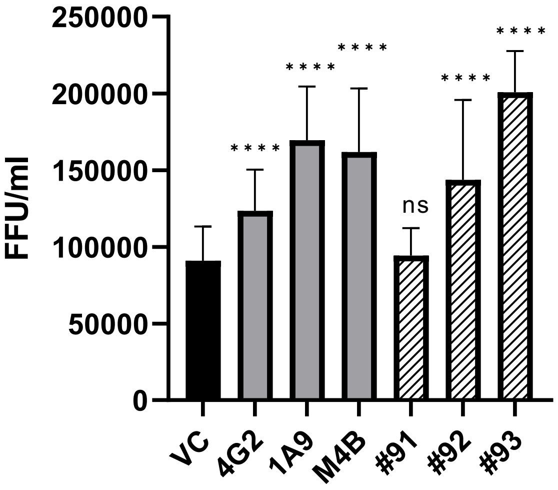

Supplementary Figure S8: Replicating virus in supernatants from ADE of dengue by the antibodies and convalescent plasma samples against SARS-CoV-2 in K562 cells. Replicating DENV2 was quantitated as foci forming units/ml (FFU/ml) of the supernatants of ADE assay performed in K562 cells. Serial 10-fold dilutions of the supernatant was used to infect the Vero E6 cells and FFU/ml was calculated 48 h post-infection. Statistical significance was calculated using One-way ANOVA followed by Dunnett's test. Asterisk (*) indicates statistically significant difference between the virus control and treated samples. P value = 0·1234 (ns), 0·0332 (*), 0·0021 (**), 0·0002 (***), <0·0001 (****).

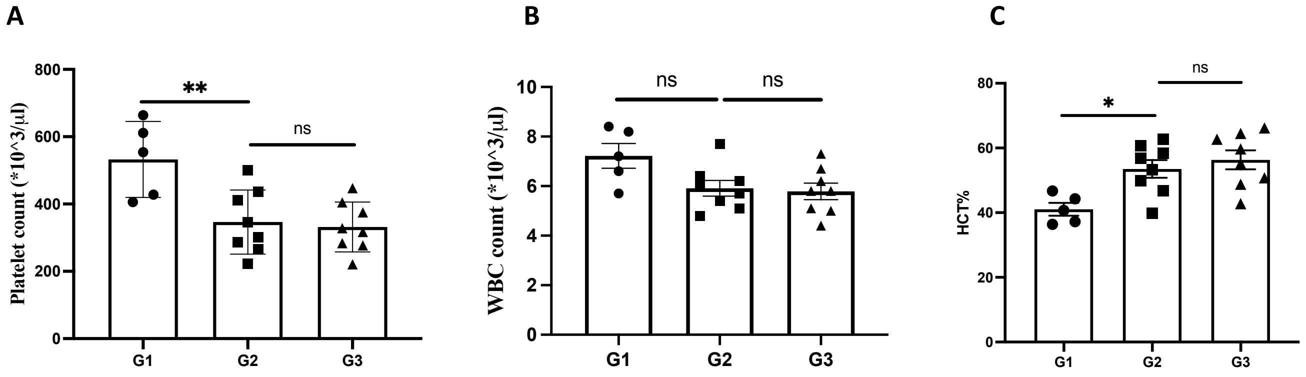

Supplementary Figure S9. Haematological analysis of dengue-infected AG129 mice following SARS-CoV-2 challenge. Prefilling blood samples were analyzed for (a) platelet count, (b) WBC count, and (c) haematocrit percentage. Data are presented as mean ± SEM; *P < 0.05, **P < 0.01, ns = not significant.

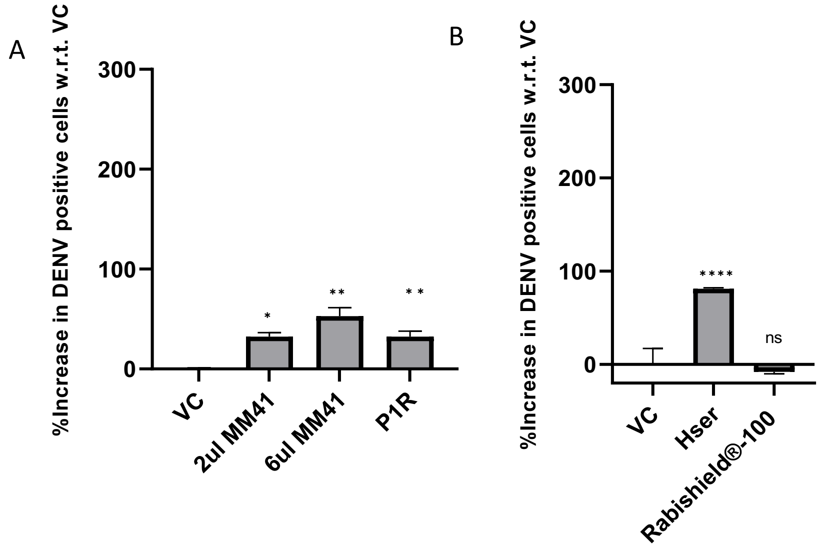

Supplementary Figure S10: ADE of dengue virus infection by the monoclonal and polyclonal antibodies and serum against SARS-CoV-2 in U937 cells.

ADE due to (A) 2µl and 6µl of anti-Spike monoclonal antibody MM41 and polyclonal antibody raised in rabbit, P1R, (B) Hamster serum immunized with SARS-CoV-2 spike and negative control (Rabishield®). Statistical significance was carried out using One-way ANOVA followed by Dunnett's test. Asterisk (*) indicates that difference between the virus control and the serum samples is statistically significant ($p < 0{\cdot}0001$). P value = 0·1234 (ns), 0·0332 (*), 0·0021 (**), 0·0002 (***), <0·0001 (****).

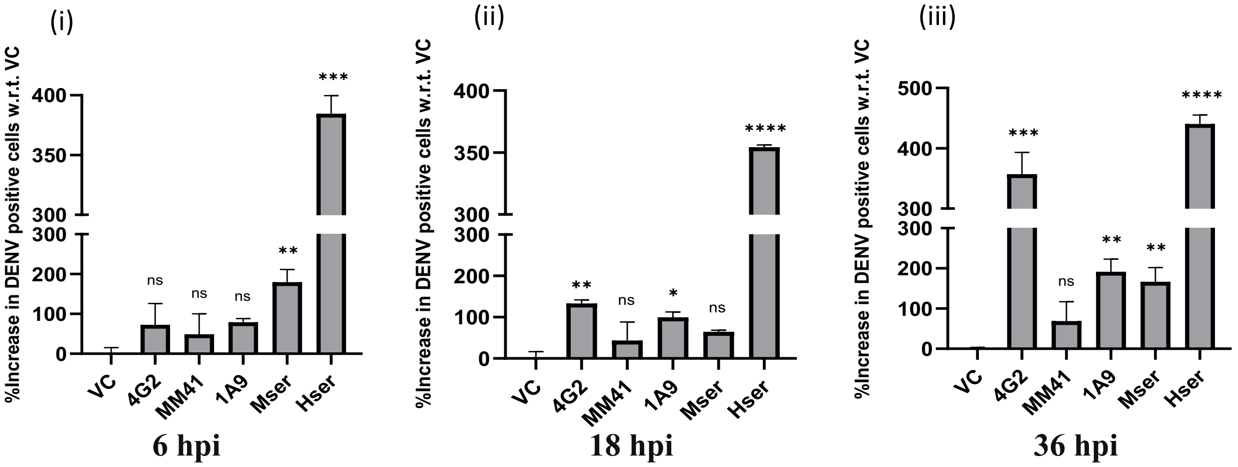

Supplementary Figure S11: Time point-based kinetics of ADE of dengue virus infection by the SARS-CoV-2 anti-Spike monoclonal antibodies and sera raised in mice and hamsters against Spike. ADE assay was performed in which post-incubation of antibody and DENV-2, the percentage increase in the DENV-2 positive cells was measured with respect to the virus-only control using flowcytometry. 4G2 was used as a positive Control. (i) ADE due to anti-Spike monoclonal antibodies and animal sera raised against SARS-CoV-2 spike at 6 hpi (ii) 18 hpi, and (iii) 36 hpi. Error bars indicate the standard deviation of each sample performed in duplicates. Statistical significance was carried out using One-way ANOVA followed by Dunnett's test. Asterisk (*) indicates that difference between the virus control and the serum samples is statistically significant ($p < 0{\cdot}0001$). P value = 0·1234 (ns), 0·0332 (*), 0·0021 (**), 0·0002 (***), <0·0001 (****).

1. Ku Z, Xie X, Lin J, et al. Engineering SARS-CoV-2 specific cocktail antibodies into a bispecific format improves neutralizing potency and breadth. *Nat Commun*. Sep 22 2022;13(1):5552. doi:10.1038/s41467-022-33284-y
2. Zhang L, Cao L, Gao XS, et al. A proof of concept for neutralizing antibody-guided vaccine design against SARS-CoV-2. *Natl Sci Rev*. Aug 2021;8(8):nwab053. doi:10.1093/nsr/nwab053
3. Liu Y, Soh WT, Kishikawa JI, et al. An infectivity-enhancing site on the SARS-CoV-2 spike protein targeted by antibodies. *Cell*. Jun 24 2021;184(13):3452-3466 e18. doi:10.1016/j.cell.2021.05.032
4. Yang M, Li J, Huang Z, et al. Structural Basis of a Human Neutralizing Antibody Specific to the SARS-CoV-2 Spike Protein Receptor-Binding Domain. *Microbiol Spectr*. Oct 31 2021;9(2):e0135221. doi:10.1128/Spectrum.01352-21
5. Shan S, Mok CK, Zhang S, et al. A Potent and Protective Human Neutralizing Antibody Against SARS-CoV-2 Variants. *Front Immunol*. 2021;12:766821. doi:10.3389/fimmu.2021.766821
6. Tortorici MA, Beltramello M, Lempp FA, et al. Ultrapotent human antibodies protect against SARS-CoV-2 challenge via multiple mechanisms. *Science*. Nov 20 2020;370(6519):950-957. doi:10.1126/science.abe3354
7. Dussupt V, Sankhala RS, Mendez-Rivera L, et al. Low-dose in vivo protection and neutralization across SARS-CoV-2 variants by monoclonal antibody combinations. *Nat Immunol*. Dec 2021;22(12):1503-1514. doi:10.1038/s41590-021-01068-z
8. Ozawa T, Tani H, Anraku Y, et al. Novel super-neutralizing antibody UT28K is capable of protecting against infection from a wide variety of SARS-CoV-2 variants. *MAbs*. Jan-Dec 2022;14(1):2072455. doi:10.1080/19420862.2022.2072455
9. Takeshita M, Fukuyama H, Kamada K, et al. Potent SARS-CoV-2 neutralizing antibodies with therapeutic effects in two animal models. *iScience*. Dec 22 2022;25(12):105596. doi:10.1016/j.isci.2022.105596
10. Zhou B, Zhou R, Tang B, et al. A broadly neutralizing antibody protects Syrian hamsters against SARS-CoV-2 Omicron challenge. *Nat Commun*. Jun 23 2022;13(1):3589. doi:10.1038/s41467-022-31259-7
11. Parzych EM, Du J, Ali AR, et al. DNA-delivered antibody cocktail exhibits improved pharmacokinetics and confers prophylactic protection against SARS-CoV-2. *Nat Commun*. Oct 6 2022;13(1):5886. doi:10.1038/s41467-022-33309-6
12. Ren P, Hu Y, Peng L, et al. Function and Cryo-EM structures of broadly potent bispecific antibodies against multiple SARS-CoV-2 Omicron sublineages. *bioRxiv*. Mar 15 2023;doi:10.1101/2022.08.09.503414
13. Yuan M, Wu NC, Zhu X, et al. A highly conserved cryptic epitope in the receptor binding domains of SARS-CoV-2 and SARS-CoV. *Science*. May 8 2020;368(6491):630-633. doi:10.1126/science.abb7269
14. Callaway HM, Zyla D, Larrous F, et al. Structure of the rabies virus glycoprotein trimer bound to a prefusion-specific neutralizing antibody. *Sci Adv*. Jun 17 2022;8(24):eabp9151. doi:10.1126/sciadv.abp9151
15. Rouvinski A, Guardado-Calvo P, Barba-Spaeth G, et al. Recognition determinants of broadly neutralizing human antibodies against dengue viruses. *Nature*. Apr 2 2015;520(7545):109-13. doi:10.1038/nature14130
